# An ontology-based method for assessing batch effect adjustment approaches in heterogeneous datasets

**DOI:** 10.1093/bioinformatics/bty553

**Published:** 2018-09-08

**Authors:** Florian Schmidt, Markus List, Engin Cukuroglu, Sebastian Köhler, Jonathan Göke, Marcel H Schulz

**Affiliations:** 1 Max Planck Institute for Informatics, Saarland Informatics Campus, Saarbrücken, Germany; 2 Cluster of Excellence MMCI, Saarland University, Saarland Informatics Campus, Saarbrücken, Germany; 3 Graduate School of Computer Science, Saarland Informatics Campus, Saarbrücken, Germany; 4 Genome Institute of Singapore, Computational Genomics and Transcriptomics, Singapore; 5 Chair of Experimental Bioinformatics, TUM School of Life Sciences, Technical University of Munich, Freising, Germany; 6 Berlin Institute of Health (BIH), Berlin, Germany; 7 Institute for Cardiovascular Regeneration, Goethe University, Frankfurt am Main, Germany; 8 German Center for Cardiovascular Research, Partner Site Rhein-Main, Frankfurt am Main, Germany

## Abstract

**Motivation:**

International consortia such as the Genotype-Tissue Expression (GTEx) project, The Cancer Genome Atlas (TCGA) or the International Human Epigenetics Consortium (IHEC) have produced a wealth of genomic datasets with the goal of advancing our understanding of cell differentiation and disease mechanisms. However, utilizing all of these data effectively through integrative analysis is hampered by batch effects, large cell type heterogeneity and low replicate numbers. To study if batch effects across datasets can be observed and adjusted for, we analyze RNA-seq data of 215 samples from ENCODE, Roadmap, BLUEPRINT and DEEP as well as 1336 samples from GTEx and TCGA. While batch effects are a considerable issue, it is non-trivial to determine if batch adjustment leads to an improvement in data quality, especially in cases of low replicate numbers.

**Results:**

We present a novel method for assessing the performance of batch effect adjustment methods on heterogeneous data. Our method borrows information from the *Cell Ontology* to establish if batch adjustment leads to a better agreement between observed pairwise similarity and similarity of cell types inferred from the ontology. A comparison of state-of-the art batch effect adjustment methods suggests that batch effects in heterogeneous datasets with low replicate numbers cannot be adequately adjusted. Better methods need to be developed, which can be assessed objectively in the framework presented here.

**Availability and implementation:**

Our method is available online at https://github.com/SchulzLab/OntologyEval.

**Supplementary information:**

Supplementary data are available at *Bioinformatics* online.

## 1 Introduction

A growing number of international consortia such as The Cancer Genome Atlas (TCGA), the Genotype-Tissue Expression (GTEx) project and the International Human Epigenome Consortium (IHEC) have generated a wealth of epigenomic profiling data of cell lines, sorted primary cells and tissue samples. These data will be of tremendous help in unraveling mechanisms of cell differentiation and in identifying patterns of epigenetic dysregulation in various diseases. A number of studies have shown that joint analysis of data from multiple projects enable novel applications of biological relevance (Cao *et al.*, 2017; Zang *et al.*, 2016). However, these integrative analyses are often hampered by batch effects, i.e. variation between datasets that is of technical origin and does not reflect biological variation. A common example of a batch effect is that in principal component analysis (PCA), samples often cluster by laboratory, processing day or experimental protocol rather than by their biological characteristics. Ignoring batch effects can lead to false conclusions as it has been shown for many large-scale projects in genomic research (Leek *et al.*, 2010) and it is now commonly accepted that batch effects should not be ignored in data analysis (Goh *et al.*, 2017).

Various approaches have been developed for batch effect adjustment (BEA) (Jacob *et al.*, 2016; Johnson *et al.*, 2007; Leek and Storey, 2007) and are routinely applied in genomics research [see Lazar *et al.* (2013) for a comprehensive review of available methods]. Selecting the best method for a given dataset is not straightforward, in particular when the dataset shows large sample heterogeneity and low replicate numbers. Within IHEC for example, the individual contributing projects have a different biological focus. Even in the absence of batch effects, we would thus expect samples to cluster mostly by project in PCA. Unfortunately there are very few instances where sample types, i.e. samples of the same cell line, cell type or tissue, have been included in more than one project.

In this study, we are interested in learning how methods for BEA can be compared best and to understand where methods fail in such difficult application scenarios. A common approach for assessing BEA performance is visual inspection in reduced dimensions (PCA, t-SNE) before and after BEA. However, we and others find this visual inspection to be highly subjective and non-interpretable, especially if the batch is not associated to the highest variance present in the data (Reese *et al.*, 2013). In the following, we briefly discuss quantitative alternatives and outline why they are not suitable for assessing BEA on heterogeneous datasets with low replicate numbers, before we present and evaluate an alternative approach based on leveraging information from an ontology.

A common way to assess batch adjustment performance quantitatively is to determine the overlap of samples from different studies before and after analysis using their distance in an n-dimensional space, e.g. by computing the ratio of samples belonging to the same study and those belonging to a different study for the k-nearest neighbors of each sample [known as mixture score (Lazar *et al.*, 2013)].

Pearson correlation of replicates can be informative of BEA performance, i.e. if the correlation of replicates is expected to improve through BEA. However, for many of the samples in IHEC few or no replicates are available, making this type of analysis challenging. Alternatively, a classifier can be trained to predict the group variables, like cell types or tissues. Successful BEA is then expected to lead to better classification performance on test data not seen during the training phase. However, this approach cannot be used as an indicator of data quality since the low replicate number does not allow for splitting the data into training and test data (Luo *et al.*, 2010).

A different approach is to measure how skewed a gene’s distribution is across studies, e.g. by evaluating the skewness of the cumulative density function (Lazar *et al.*, 2013). However, considering that gene expression profiles between vastly different sample types is expected to show considerable variability for most genes, this approach is not always suitable. The same argumentation rules out the use of differential gene expression analysis as suggested in some studies (Gagnon-Bartsch and Speed, 2012; Leek and Storey, 2007) or testing for the uniform *P*-value distribution of control genes (Leek and Storey, 2007).

Another promising strategy is principal variance component analysis (Chen *et al.*, 2011; Leek *et al.*, 2010), which first extracts principal components and uses these in variance component analysis to understand the influence of known batch variables. After BEA, the contribution of known batch variables is expected to be lower or even non-existent. Unfortunately for many projects, only few potential sources of batch effects are documented in the meta data. Moreover, potential batch variables such as laboratory or sample preparation protocol correlate strongly with the sample type, making principal variance component analysis unfeasible. A recently published approach based on probabilistic principal component and covariate analysis (PPCCA) attempts to circumvent some of these issues by incorporating covariates into a PCA and performing statistical tests for each principle component separately to check whether it contains a batch effect (Nyamundanda *et al.*, 2017). However, this approach also requires that covariates are known beforehand and thus does not allow quantifying whether the BEA improved the data at hand or not.

The vast sample numbers available in single cell RNA-seq technology further motivated the development of a *χ*^2^-based method specialized for this type of data (Buttner *et al.*, 2017).

Overall, BEA carries the risk of removing biologically relevant group differences, in particular when sample groups are not evenly distributed between batches as is the case for many large-scale datasets including TCGA, GTEx and IHEC (Lazar *et al.*, 2013; Nygaard *et al.*, 2016). This led us to the question how the performance of BEA methods could be assessed and compared objectively in a heterogeneous scenario with few replicates and many diverse sample types.

## 2 Approach

The established methods for assessing BEA performance consider only the similarity of samples belonging to the same sample type. However, if we can quantitatively describe the expected similarity between sample types, we gain the ability to compare one sample against all others. For example, we can then consider that a liver cell may be expected to be more similar in expression to a kidney cell than to a brain cell.

**Fig. 1.**
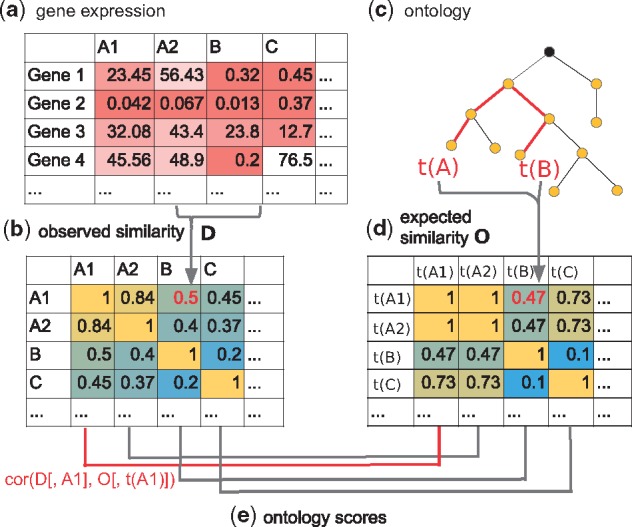
Overview of the method. (**a**) Given a gene expression matrix as input, we compute similarities for all pairs of samples, giving rise to (**b**) a matrix *D* of observed similarities. (**c**) Using an ontology as input, we compute a matrix *O* of (**d**) expected similarities based on the path lengths between the terms corresponding to each sample in (c). Finally, we correlate for each sample two vectors, namely the observed sample similarities from (b) to the expected similarities in (d) that correspond to their sample type. This yields (**e**) ontology scores for each sample in (a)

This led us to the *Cell Ontology* (Bard *et al.*, 2005), an established hierarchical description of individual cell types. We used the *Cell Ontology* to leverage previous knowledge to derive an estimate of expected sample type similarity. Our method, which is depicted in Figure 1, extracts three ordered vectors for each sample: (i) from the ontology, we extract the expected similarity of the chosen sample to all other samples, (ii) we compute the similarity of this sample to all other samples, (iii) we recompute the similarity in (ii) after BEA. Finally, we correlate this to the observed distances before (ii) and after (iii) BEA and refer to this as the *ontology score.*

Our null hypothesis is that BEA does not lead to significantly higher correlation of expected and observed similarity, i.e. the ontology score will not improve.

We systematically investigate the extent of batch effects between ENCODE (Dunham *et al.*, 2012), Roadmap (Kundaje *et al.*, 2015), BLUEPRINT (Adams *et al.*, 2012) and DEEP (http://www.deutsches-epigenom-programm.de/) gene expression (RNA-seq) data. Additionally, we consider a less diverse set comprised of uniformly processed data from TCGA (Weinstein *et al.*, 2013) and GTEx (Consortium *et al.*, 2015).

## 3 Materials and methods

### 3.1 Data

#### *3.1.1* IHEC data

We downloaded FASTQ files for 36 ENCODE and 112 Roadmap RNA-Seq experiments from the ENCODE web portal. Furthermore, we obtained FASTQ files for 12 samples from the DEEP data portal and for 56 samples from the BLUEPRINT data portal. Gene expression is quantified in *transcripts per million (TPM)* with Salmon (v. 0.8.2) using reference transcript sequences from Gencode Release v26 (GRCh38.p10). ENCODE accession numbers, DEEP and Blueprint sample IDs, tissue/cell-type assignments as well as sample number per consortia are listed in Supplementary Tables S1 and S2, respectively.

#### *3.1.2* GTEx and TCGA data

FASTQ files for 6575 RNA-Seq samples were downloaded from the GTEx project (Consortium *et al.*, 2015). 741 RNA-Seq samples were downloaded from the TCGA project (Weinstein *et al.*, 2013). Note that we only include TCGA control samples, i.e. tumor-adjacent tissue samples, while tumor samples are neglected. For the purpose of this study, we focus on the five tissues that are covered in both GTEx and TCGA: colon, liver, kidney, prostate and thyroid amounting to 1062 GTEx and 274 TCGA samples. Sample identifiers and sample number per tissue are provided in Supplementary Table S3.

RNA-Seq data is mapped against the human genome version hg19 with TopHat2 (v.2.0.12) (Kim *et al.*, 2013) using the Ensembl v75 gene annotation. R-3.4.1 (Team, 2014) and Bioconductor 3.0 (Gentleman *et al.*, 2004) are used for the RNA-Seq analysis.

Mapped reads are counted using the R package Genomic Alignments (Lawrence *et al.*, 2013) using the parameters *mode = Union*, *inter.feature = FALSE.* Only primary read alignments are retained. Sample normalization values of the counts are calculated using DESeq2 (Love *et al.*, 2014).

### 3.2 Assessing the similarity of samples using an ontology based distance matrix and gene expression data

Figure 1 provides an overview of the steps involved in computing the ontology score. Details are provided in the following section.

#### *3.2.1* Inferring expected sample similarities from the *cell ontology*

Between all terms included in the *Cell Ontology* (Bard *et al.*, 2005), we compute a pairwise similarity *sim*(*t_i_*, *t_j_*) between terms *t_i_* and *t_j_* using both Jaccard coefficients (*sim_jac_*) and cosine similarity (*sim_cos_*) (Pesquita *et al.*, 2009). The function *A*(*t_i_*) returns the set of ancestors for a given term in the cell ontology. Note that *A*(*t_i_*) contains *t_i_* as well and that only subclass relationships are considered. For the cosine similarity we obtain a vector representation $v_{t}$ for a term *t.* The vector has |CL | entries, where |CL | is the number of terms in the *Cell Ontology* and each entry corresponds to one term. We set every entry in $v_{t}$ to zero and every entry that corresponds to an entry in *A*(*t*) to one. The Jaccard similarity and cosine similarity are then defined as

(1) $${\text{sim}}_{\text{cos}}(t_{i},t_{j})=\frac{v_{t_{i}}\cdot v_{t_{j}}}{\sqrt{v_{t_{i}}^{2}}\cdot \sqrt{v_{t_{j}}^{2}}},\;{\text{sim}}_{\text{jac}}(t_{i},t_{j})=\frac{A(t_{i})\cap A(t_{j})}{A(t_{i})\cup A(t_{j})}.$$

An example is provided in Supplementary Figure S13. We manually map all IHEC, GTEx and TCGA samples to *Cell Ontology* terms. Using the entire set of similarities for all terms and the manually generated sample-term mappings, we can generate matrices holding pairwise sample similarities *sim*(*s_k_*, *s_l_*) for any combination of samples by considering the respective similarity measures *sim* and sample types $t_{s_{k}}$ and $t_{s_{l}}$ in the ontology. We calculate $sim(s_{k},s_{l})=sim(t_{s_{k}},t_{s_{l}})$. Thereby we obtain symmetric similarity matrices *O_jac_* and *O_cos_.* We point out that these similarity matrices can be easily converted to distance matrices by $dist(s_{k},s_{l})=1-sim(t_{s_{k}},t_{s_{l}})$.

#### *3.2.2* Computing a sample similarity matrix with respect to gene expression data using principal component analysis (PCA)

In order to generate a gene expression-based distance matrix for all samples, we run a PCA on a full RNA-seq dataset of interest, thereby reducing its dimensionality. The first Principal Components (PCs) capture most of the variability between the samples, thus they can be used to distinguish the samples from each other. Using the first four PCs (explaining between 86–95*%* of variance) we compute a sample similarity matrix *D* based on Spearman correlation.

#### *3.2.3* Comparing expression-based distances with expected ontology distances

We construct *O* such that it has the same dimensionality as *D*, i.e. for each sample *k* the observed similarities *D*[, *k*] match the expected similarities *s_k_* in *O*[, *k*]. This allows for assessing how well the expected similarities *O* fit to the inferred similarities *D* through a global score based on the similarity of the two matrices, e.g. via the inner product. However, to avoid losing sample-specific information, we instead chose to compute a vector *u* of *ontology scores* with

(2) $$u_{k}=cor(D[,k],O[,k])$$

for each sample *k* and *cor* as either Spearman *S_sp_* or Pearson *S_p_* correlation. Note that all figures presented in this manuscript are based on *O_cos_* and *S_sp_.* For results on alternative combinations we refer to the Supplementary Material. Using per sample scores allows us to investigate effects at different granularity, reaching from individual samples to groups of samples pertaining to specific batches (e.g. comparing consortia) or group variables (e.g. comparing tissues).

We test different combinations of similarity measures for computing *D*, *O* and *u* and find that the differences between the scoring schemes are marginal. Hence, the overall conclusions drawn from the scores are invariant to the used scoring scheme.

### 3.3 Correcting for batch effects

We use three widely used BEA methods considered in this work, namely *Combat* (Johnson *et al.*, 2007), *SVA* (Leek and Storey, 2007) and *RUV* (Jacob *et al.*, 2016) which are briefly described here. Please refer to the respective publications for a detailed description of the methods. The most common strategy in BEA is to transform data from different batches to have the same or similar mean and variance for each gene. *Combat* is a widely used method that infers gene-scaling parameters robustly by pooling information across genes with similar expression profile using an empirical Bayes approach (Johnson *et al.*, 2007).

*Combat* was originally designed to adjust batch effects in microarray datasets with small batch sizes in mind. It can be used with or without adding group variables whereas the batch variables have to be provided. Another strategy for BEA is matrix factorization. Here, we assume that most of the variance in the raw data is due to batch effects. Thus, batch variables are expected to correlate with one or several of the first factors. These batch-associated factors are identified and removed from the data.

A popular example for this strategy is surrogate variable analysis (*SVA*) (Leek and Storey, 2007). *SVA* estimates the number of latent variables to be removed and can be run with or without adding group variables to the model.

Remove unwanted variation (*RUV*) is an alternative approach that uses a set of control genes to identify factors associated with batch effects (Jacob *et al.*, 2016). While *RUV* can infer suitable control genes from the data, a more common strategy is to use a set of housekeeping genes that are expected to show little variance within a batch. *RUV* removes the variation derived from the control genes via linear regression. In contrast to *Combat* and *SVA*, neither group nor batch variables are provided.

*Combat* and *SVA* were used through the *sva* R-package (v 3.24.4). *RUV* is applied through the *RUVNormalize* R-package (v 1.12.0). In this study, we provide *Combat* with information on the present batches, i.e. the data source, as well as the tissue assignment of the samples. In *SVA*, we define the probability for a gene *g* to be a control gene *pc_g_* as

(3) $$pc_{g}=1-\frac{rank(\sigma_{\left(g\right)})}{max(rank(\sigma_{\left(g\right)}))},$$

with *σ*_(_*_g_*_)_ being the standard deviation of the expression of gene *g* across all samples and *rank* returns the corresponding rank of *σ*_(_*_g_*_)_ compared to all genes. The motivation for this is to preferably use genes as controls that have stable expression across samples. *SVA* is also provided with the tissue to sample assignment. For *RUV*, we use a set of housekeeping genes suggested by NanoString Technologies (2009) as control genes.

## 4 Results

We developed the ontology score to assess if BEA is beneficial on heterogeneous datasets. To assess if the score fulfills this expectation, we present several analyses. First, we use randomization experiments to assess the benefit of using the *Cell Ontology* for computing ontology scores. Second, we add artificial noise to GTEx data to assess the robustness of the score. Third, we add an artificial batch effect to GTEx data to investigate if the ontology score drops as expected and to study to what extent the score can be recovered through BEA. Finally, we apply the ontology score to TCGA, GTEx and IHEC data to demonstrate what can be learned from this score when applied to batch-adjusted heterogeneous data.

### 4.1 The ontology score leverages information captured in the *cell ontology*

To learn about the robustness of the ontology score, we conduct randomization experiments using GTEx data. To this end, we generate 100 sets in which each sample is assigned a random tissue-term from the available ontology terms. For each set, we compute corresponding expected distance matrices and subsequently recompute the ontology scores.

**Fig. 2.**
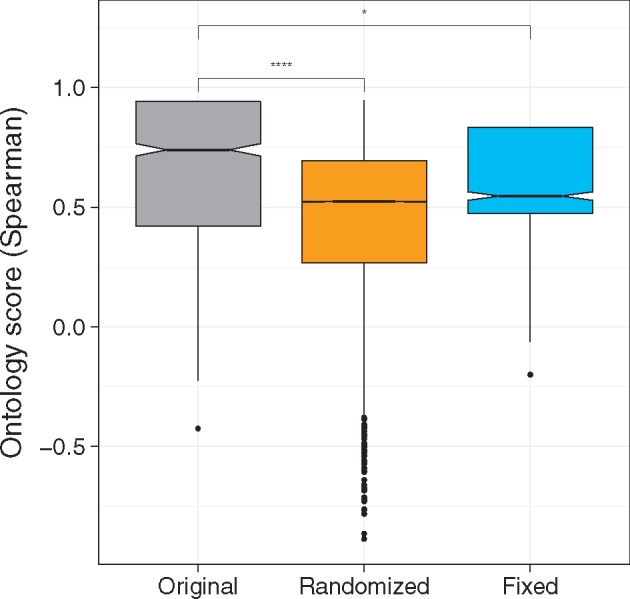
The ontology score computed on GTEx normal samples using the original ontology matrix, a fixed ontology matrix, i.e. identical tissues have a similarity of 1.0 while different tissues have a similarity of 0.25, and using randomly sampled ontology matrices (sampled 100 times from all available ontology terms). According to a Wilcoxon–Mann–Whitney test, the original ontology leads to a significantly higher score than both alternative versions (***P-value <1*e*– 4, *P-value <0.05)

As shown in Figure 2, the mean score computed based on the randomized ontology is significantly lower than the mean score of the original ontology matrix. In a more realistic scenario, we use an ontology in which samples from the same tissue are assigned a similarity of 1.0 and 0.25 otherwise. This version allows us to study if leveraging information about the similarity between sample types leads to a significantly higher score, which also holds true. Taken together, these results indicate that the ontology score indeed informs about the sample similarities we can expect from gene expression data. Moreover, it emphasizes that an accurate mapping of samples to cell ontology terms strongly influences the quality of the ontology scores, i.e. the better the ontology and the assigned sample labels reflect the underlying biology, the higher the ontology score will be.

In Supplementary Figure S1a, we show the results for the randomization experiment using other scoring schemes. Supplementary Figure S1b illustrates the score behavior individually for each tissue.

### 4.2 The ontology score is sensitive to noise in the data

**Fig. 3.**
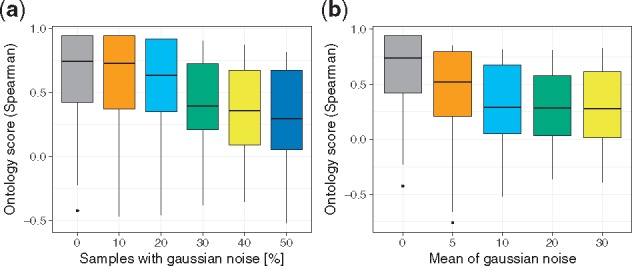
(**a**) Changes in the distribution of the ontology score depending on artificial Gaussian noise *N*(*μ* = 10, *σ* = 1) added to a subset of GTEx samples across all tissues. The fraction of samples that was contaminated with noise is shown on the x-axis. With increasing contamination, the score is dropping. (**b**) Depicts how the score is influenced by varying the mean (*μ*) of Gaussian noise added to the samples. For this experiment, noise is added to 50% of all samples across all tissues, with varying mean. With increasing noise intensity, the score drops and remains constant after *μ* > 10

Next, we conduct two simulation studies to learn about the effect of artificial noise on the ontology score. Adding artificial noise systematically can be seen as introducing an artificial batch effect. In the first simulation, we add Gaussian noise *N*(*μ* = 10, *σ* = 1) to all genes across all tissues for different fractions of GTEx samples reaching from 0 to 50*%.* As shown in Figure 3a, the score decreases when the fraction of samples exposed to noise increases. This can also be observed using alternative score variants (Supplementary Fig. S2a and b). In a second simulation, we added Gaussian noise to 50% of all samples with constant variance but increasing mean reaching from 0 to 30. As shown in Figure 3b the score is dropping rapidly with increasing noise intensity but stays rather constant after *μ* ≥ 10. Similar observations can be made with alternative scores (Supplementary Fig. S2c and d). Taken together, these results show that the ontology score quantifies increasing levels of distortion in data associated with the number of affected samples and the extent of the noise involved.

### 4.3 The performance of batch effect adjustment is captured by the ontology score

**Fig. 4.**
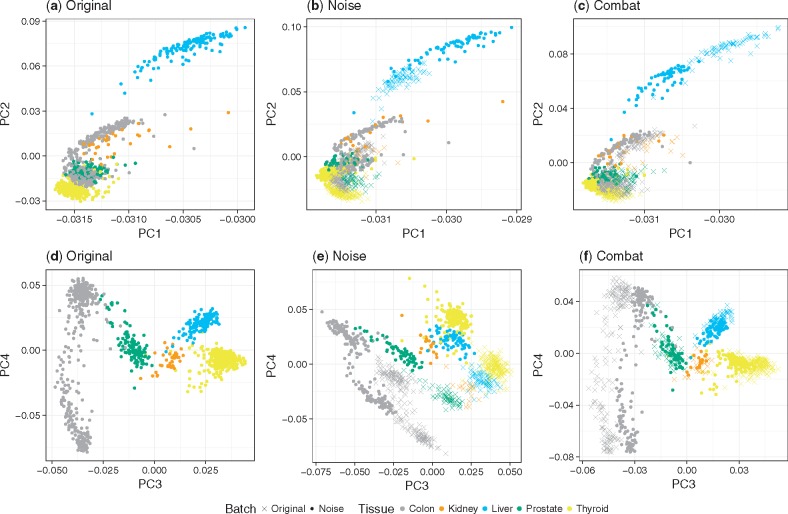
In (**a**–**c**), we show the 1*st* and 2*nd* PC, in (**d**–**f**) the 3*rd* and 4*th* PC of a PCA computed on the original GTEX data. In (b) and (e), a shift caused by the Gaussian noise is clearly visible. One can see that there are two batches per tissue and the tissue specific clustering present in (d) is largely lost. (c) and (f) illustrate the PCA space after the noisy data was adjusted with *Combat.* The shift visible in (e) disappeared in (f), and overall the plot is similar to (d), i.e. the tissue specific clustering is mostly restored. However, as shown in (c), the liver samples were not adjusted properly. A new shift, which is different from the shift caused by the Gaussian noise (b) has been introduced by *Combat.* This leads to a different clustering compared to the original data (a)

**Fig. 5.**
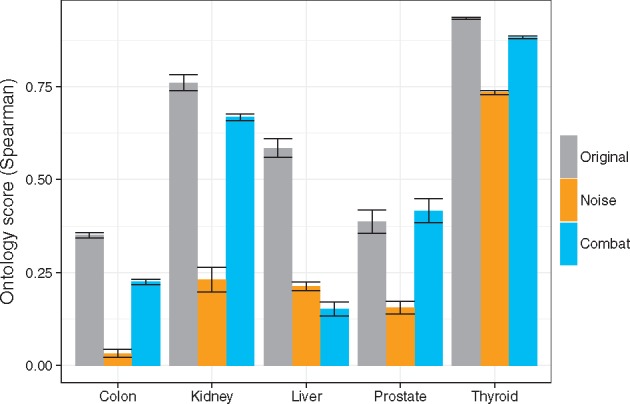
Here, we show the ontology score for individual GTEx normal tissues using three different datasets indicated by the color: (1) original GTEx data, (2) data from (1) with Gaussian noise *N*(*μ* = 10, *σ* = 1) added to 50% of samples across all tissues, and (3) data from (2) processed with *Combat.* The ontology score drops upon the addition of noise for all tissues. It can be nearly completely restored to its original value through *Combat*, except for the liver samples

We extend the simulation study introduced in the previous section by attempting to adjust for the artificial noise using *Combat* as one example of a batch adjustment method. *Combat* is, by design, well suited to adjust for the linear shift we imposed on the data. We use PCA plots to visualize the variance present in the data. PCA analysis shows that the original GTEx data clusters per tissue (Fig. 4a and d and Supplementary Fig. S3a). As expected, adding Gaussian noise to 50% of the samples leads to the formation of two large clusters in the PCA, breaking up the tissue-specific clustering of the original data (Fig. 4b and e and Supplementary Fig. S3b). Performing a PCA on data adjusted with *Combat* shows that samples do no longer cluster by batches and instead cluster by tissue as in the original data (Fig. 4c and f and Supplementary Fig. S3c). Encouragingly, the ontology score reflects these observations as well. As shown in Figure 5 (and Supplementary Fig. S4), the score decreases when noise is added and is nearly restored to the original level after adjustment via *Combat* with the notable exception of liver samples. For the latter, PC1 and PC2 show that the noise could not be properly removed (Fig. 4a and c and Supplementary Fig. S3c). Consequently, the ontology score does not improve either.

This example illustrates the applicability of our method in a controlled environment with merely five tissues and two well-separated batches. Yet it is already challenging to assess the performance and limitations of the BEA method by visual inspection of the PCA, whereas the ontology score provides a simple and effective way to circumvent this subjective assessment (more PCA plots in Supplementary Fig. S3).

### 4.4 Application to heterogeneous datasets

We use the ontology score in two use cases. (i) we combined data from TCGA and GTEx as well as (ii) data from the IHEC members Blueprint, DEEP, ENCODE and Roadmap.

**Fig. 6.**
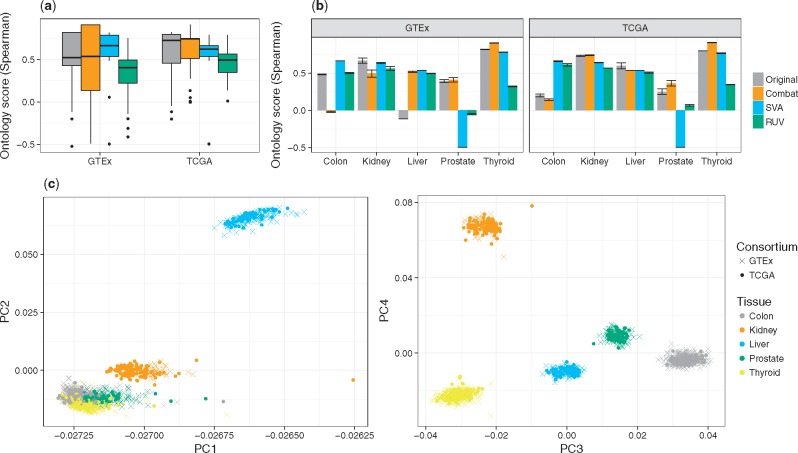
(**a**) Depicts the ontology scores computed for samples of five tissues extracted from GTEx and TCGA, before and after batch effect removal. While *SVA* seems to perform well on GTEx data, it appears to be not beneficial to apply any adjustment method at all on the TCGA data. (**b**) We observe that both *SVA* and *RUV* have issues with the Prostate data in this setting, as the ontology score is turning negative. Also, we note that the score for original liver samples from GTEx is very low. (**c**) PCA analysis of the *SVA* adjusted data showing PC1 vs. PC2 and PC3 vs. PC4. While the clustering looks very well in PC3 and PC4, we note that the prostate samples are overlapping with colon and thyroid samples, which is reflected by the negative ontology score for prostate using *SVA*

Figure 6a shows the distribution of the ontology score in the first use case. The *RUV* method does not perform well according to the ontology score, while *SVA* seems to be able to successfully adjust GTEx data, while exhibiting poor performance on TCGA data. *Combat* shows only marginal improvement in the ontology scores. Figure 6b as well as Supplementary Figure S5 highlight that a more fine-grained view is useful to learn which samples and batches benefit from BEA. Without separating ontology scores by tissues and projects little differences are revealed (Supplementary Fig. S5a). Splitting the ontology score distribution for individual tissues reveals that *SVA* introduces negative scores for prostate samples (Supplementary Fig. S5a and b). Further splitting by tissue and project reveals additional details (Fig. 6b and Supplementary Fig. S5c), e.g. that *SVA* could not improve the quality of liver data in TCGA, while it did improve it in GTEx data. Splitting up ontology scores in this fashion offers an explanation for the score difference observed between TCGA and GTEx, which are not apparent in a visual inspection of PCA plots (Supplementary Fig. S6). Moreover, the ontology score reveals potential issues not visible by PCA. For instance, Figure 6c shows *SVA* leads to a visually perfect separation of samples according to tissues whereas the ontology score suggests that the cluster with the prostate samples is not in the expected position relative to other tissue types, suggesting that *SVA* may have introduced an artifact here. Indeed, a closer investigation of the correlations of prostate samples with samples of other types confirmed this (Supplementary Fig. S11). Overall, our results suggest that BEA does not seem beneficial in this particular use case.

Additionally, we applied the ontology score in a more challenging scenario with many diverse tissues and cell types (65 in total) originating from four consortia, namely DEEP, Blueprint, Roadmap and ENCODE. Here, only few samples are available per tissue/cell-type and the overlap of tissues/cell-types being present across multiple consortia is small.

**Fig. 7.**
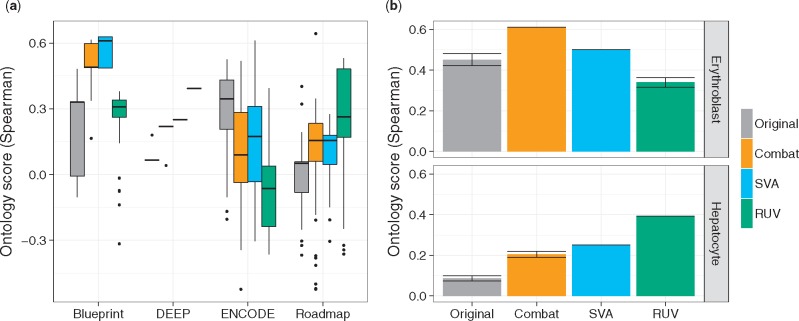
(**a**) Here we show the ontology score for adjusting batch effects in a heterogeneous dataset with 65 tissues from Blueprint, DEEP, ENCODE and Roadmap. The batch adjustment methods do not perform uniformly well across data sources, e.g. *RUV* performs favorably for DEEP and Roadmap data, while *Combat* seems to obtain good results on Blueprint samples. For ENCODE data, no adjustment method is able to improve the score. (**b**) Example for a tissue specific view on the data. While *RUV* performs well in improving hepatocyte data, it is the worst method considering erythroblasts

As shown in Figure 7a, the *RUV* method seems to perform favorably for DEEP and Roadmap data, whereas it is the worst performing method on Blueprint data. For the latter, *Combat* shows the highest increase in the ontology score. Interestingly, according to the ontology score, no method leads to an improvement on ENCODE data. Similar observation can be made for the alternative score variations (Supplementary Fig. S7). In a diverse and complicated scenario as the one presented here, a PCA analysis as shown in Supplementary Figure S8 cannot be easily interpreted, arguing for the necessity of an alternative way to assess BEA methods objectively.

Our ontology score allows a cell-type/tissue specific analysis of the data. An example is shown in Figure 7b (and Supplementary Fig. S9). While *RUV* performs well on hepatocyte data (see also Supplementary Fig. S12), it does not work well on erythroblasts. Here, the results suggest that none of the applied methods is generally applicable. Moreover, it becomes apparent that many sample types benefit from BEA with *Combat* or *SVA* while others are either unaffected or show a decrease in ontology score.

## 5 Discussion

Heterogeneous datasets such as the one produced by IHEC do not yet offer sufficient sample numbers to leverage existing BEA methods without careful considerations. A major concern is that the procedure may further reduce statistical power or, in the worst case, introduce an additional confounding variable that obscures group differences such as those between sample types. To our knowledge there is no objective approach for assessing the quality improvement of BEA on heterogeneous datasets with few sample numbers. This motivated us to develop the ontology score, which offers two major advantages in comparison to existing methodology. First, it overcomes the problem that few samples of the same type are available by comparing each sample to all other samples. This is achieved by utilizing information captured in the *Cell Ontology.* The ontology score can thus answer the question if BEA leads to a better separation of samples according to the relative similarity changes to other sample types. Second, it allows for different BEA methods to be compared against each other and to identify strengths and limitations of each approach.

To characterize the ontology score we used GTEx gene expression data from five different tissues. Our results show that using the *Cell Ontology* leads to significantly higher scores than a randomized assignment of labels or an ontology in which we only recognize sample type identity but use a default for all other sample types. While this is encouraging and demonstrates that the ontology score works as expected, it should be noted that the ontology score is also limited by the quality of the ontology. Indeed it is not likely that biological reality can be fully captured in an ontology as previous studies have already shown. For instance, cell differentiation during hematopoiesis is more complex than previously believed (Farlik *et al.*, 2016). This may also explain why only some sample types generally benefit from BEA. While this could also be attributed to data quality, it is plausible that the ontology does not capture the similarities of these sample types adequately.

Moreover, we point out that it is not clear when a good ontology score is reached. In fact, the ontology score can only be used for relative comparisons, e.g. between original and batch-adjusted data or between datasets adjusted through different methods.

Finally, the quality of the results depends on the assignments between sample type labels and the ontology terms. We did this in a manual, error-prone fashion and learned in the process that it is not always straightforward to find the correct assignment. We thus encourage consortia to incorporate expert-curated mappings to ontology terms in their meta data.

We further study how the ontology score is affected by adding varying degrees of noise to the data. Here, the ontology score is reduced as expected, highlighting that it may also be used for assessing the performance of normalization methods (as illustrated in Supplementary Fig. S10). On the GTEx analysis we could illustrate that the ontology score can be used to isolate the effect of BEA to different groups of samples and to reveal more nuanced issues.

Another possible concern is the influence of sample numbers. In the GTEx data this did not seem to play a major role. For instance, thyroid and colon are represented through large sample numbers, yet thyroid samples achieved far higher scores than colon samples.

When applying *SVA*, *Combat* and *RUV* to a mixed dataset of GTEx and TCGA data, the ontology score suggests that BEA was generally not beneficial. The reason for this could be that TCGA control samples, which are collected in the vicinity of a tumor, are markedly different from non-cancerous tissue samples (Huang *et al.*, 2016). Nevertheless, a closer look at the ontology score yields important insights into limitations of the different BEA methods. For example, visual inspection of PCA plots shows that all tissues cluster seemingly perfect after *SVA* batch adjustment. However, the ontology score reveals that prostate samples do now show a negative correlation with the expected similarities, indicating that *SVA* may have isolated the different sample types perfectly, while removing important information that characterize group differences. This is not surprising, since *SVA* attempts to maximize the differences between the sample types. This means that the ontology score, by leveraging the additional information provided by the ontology, can be used to test whether a BEA method removed important group characteristics. By considering how relative similarities to other sample types are affected, the ontology score gains an advantage over alternative methods such as the mixture score, which only test if individual sample types cluster well. Considering only the latter would not indicate an issue in this case.

The IHEC data is the most heterogeneous example we consider in this study with very few samples overlapping between different projects. Blueprint, DEEP and ENCODE samples, which consist mainly of primary cells, benefit little from BEA. Roadmap samples, which are mostly obtained from tissues, show a more pronounced improvement depending on the method used. Here, we speculate that BEA may be more meaningful for tissue samples in which a heterogeneous mix of different cell types is distorting the results. Removal of a latent variable representing, for instance, the contribution of immune cells to a tissue sample, could explain the increase in the ontology score. Primary cells, on the other hand, are already purified and do thus not benefit from this procedure.

*SVA* and *Combat* often outperformed *RUV*, likely because they received the sample type labels as input. The poor performance of the *RUV* method for GTEx and TCGA data, may be due to our choice of housekeeping genes. On the other hand, we find examples where *RUV* leads to considerably higher ontology scores than all other methods, e.g. in DEEP and Roadmap samples, where it improved the clustering of hepatocyte and kidney samples considerably (Supplementary Fig. S12).

For the future, we see a demand for the development of novel BEA methods that can accommodate the heterogeneity observed in large international consortia like IHEC. We note that this task could be tremendously simplified by including a set of reference samples such as a panel of cell lines into each batch of a project and recommend considering this for future studies. Already today, empirical Bayes methods exist that can utilize such reference samples even when batches are otherwise unbalanced with respect to their sample types (Walker *et al.*, 2008). In absence of this, we could imagine that the expected similarity we utilize here for benchmarking could also be utilized in a batch adjustment method. Obviously, following this approach would then limit the applicability of the ontology score and raise even stronger concerns about exaggerated confidence in group differences.

Another major challenge in batch effect analysis is the lack of meta data annotation, which forces data analysts to rely on surrogate variables such as the processing date. This has been realized and efforts for harmonizing such data in IHEC are currently under way.

Here, we use RNA-seq data as the most widespread data source used in genomics studies. However, batch effects are a concern in all types of genomic and epigenomic data (Akulenko *et al.*, 2016). We will thus investigate how BEA methods perform on other types of epigenomic data in the future.

Finally, we envision that the availability of multi-omics data of the same samples, as is the case for IHEC, offers the opportunity to develop BEA methods that adjust for batch effects in a joint model that borrows information from other data types (Zang *et al.*, 2016).

## 6 Conclusion

In recent years, more and more emphasis has been put on collaborative efforts for generating molecular profiling data. While many genomics projects handle impressive sample numbers, this is not yet the case for relatively new technologies allowing to study the epigenome. Moreover, many projects focus on a particular condition, such as cancer, whereas the data available through the IHEC consortium is very heterogeneous and includes cell lines, primary cells and tissues for samples from healthy donors as well as samples from patients carrying various diseases.

While it is always preferable to include batch variables directly in statistical analysis, many downstream analysis tools do not offer this possibility. This leads to the requirement of BEA as an essential part of integrative analysis. However, it is currently unclear how useful established methods are on epigenetic data, in particular considering that these methods have been developed with larger sample numbers and balanced datasets in mind in which all sample types are included in all batches.

The ontology score we propose here is, to our knowledge, the first to robustly assess the performance of BEA, in particular when biologically relevant group variables such as the sample type are part of the adjustment procedure. Moreover, this approach is not limited to batch adjustment but can also be used to show if data normalization leads to improved results.

## Supplementary Material

Supplementary Data
