# Supplementary material for: An ontology-based method for assessing batch effect adjustment approaches in heterogeneous datasets
Source: Bioinformatics. 2018 Sep 8;34(17):i908–16. doi: 10.1093/bioinformatics/bty553 (PMC6129283; doi:10.1093/bioinformatics/bty553)
Supplement: Supplementary Data [file bty553_supplementary_material.pdf]

Supplementary Material

1. Data

Sample/Experiment ID	Consortia	Tissue	Cell Ontology Term
41_Hf01_LiHe_Ct	DEEP	Hepatocyte	CL:0000182166
41_Hf02_LiHe_Ct	DEEP	Hepatocyte	CL:0000182166
41_Hf03_LiHe_Ct	DEEP	Hepatocyte	CL:0000182166
41Hm09_LiHe_Ct	DEEP	Hepatocyte	CL:0000182166
41_Hm16_LiHe_Ct	DEEP	Hepatocyte	CL:0000182166
41_Hm25_LiHe_Ct	DEEP	Hepatocyte	CL:0000182166
41_Hf05_LiHe_St	DEEP	Hepatocyte	CL:0000182166
41_Hf11_LiHe_St	DEEP	Hepatocyte	CL:0000182166
41_HF14_LiHe_St	DEEP	Hepatocyte	CL:0000182166
41_Hf17_LiHe_St	DEEP	Hepatocyte	CL:0000182166
41_Hm07_LiHe_St	DEEP	Hepatocyte	CL:0000182166
41_Hm08_LiHe_St	DEEP	Hepatocyte	CL:0000182166
ENCSR000EYS	ENCODE	Endothelial cell of umbilical vein	CL:00026182022
ENCSR000CPK	ENCODE	keratinocyte	CL:0000312254
ENCSR000CPI	ENCODE	keratinocyte	CL:0000312254
ENCSR000EYT	ENCODE	keratinocyte	CL:0000312254
ENCSR000COO	ENCODE	fibroblast of lung	CL:00025531958
ENCSR000CPM	ENCODE	fibroblast of lung	
ENCSR000COP	ENCODE	Foreskin fibroblast	CL:10016081592
ENCSR000CTV	ENCODE	B cell	CL:0000236210
ENCSR000CUC	ENCODE	CD14-positive monocyte	CL:00010541126
ENCSR444WHQ	ENCODE	Skeletal muscle myoblast	CL:0000515430
ENCSR000CUA	ENCODE	Hematopoietic multipotent progenitor cell	CL:0000837754
ENCSR797BPP	ENCODE	Fibroblast of arm	CL:20000151188
ENCSR233IJT	ENCODE	Astrocyte	CL:0000127114
ENCSR276MMH	ENCODE	Adrenal gland	CL:10016011602
ENCSR801MKV	ENCODE	Adrenal gland	CL:10016011602
ENCSR954PZB	ENCODE	Adrenal gland	CL:10016011602
ENCSR532LJV	ENCODE	Thyroid gland	CL:00022581668
ENCSR023ZXN	ENCODE	Thyroid gland	CL:00022581668
ENCSR653ZJF	ENCODE	Transverse colon	CL:1000283774
ENCSR630VJN	ENCODE	Transverse colon	CL:1000283774
ENCSR800WIY	ENCODE	Transverse colon	CL:1000283774
ENCSR967JPI	ENCODE	Gastrocnemius medialis	CL:0000188171
ENCSR071ZLM	ENCODE	Uterus	CL:00021491532
ENCSR113HQM	ENCODE	Uterus	CL:00021491532
ENCSR042GYH	ENCODE	Ovary	CL:00020941469
ENCSR029KNZ	ENCODE	Testis	CL:00022381649
ENCSR701TST	ENCODE	Prostate gland	CL:20000591211
ENCSR968WKR	ENCODE	Bipolar spindle neuron	CL:000010390
ENCSR908ZAS	ENCODE	Hepatocyte	CL:0000182166
ENCSR828TEI	ENCODE	Myotube	CL:00023721780
ENCSR244ISQ	ENCODE	Neural progenitor cell	CL:000004739
ENCSR000EYP	ENCODE	H1-hESC	CL:000003429
ENCSR000COU	ENCODE	H1-hESC	CL:000003429

ENCSR000COW	ENCODE	H1-hESC	CL:000003429
ENCSR000COV	ENCODE	H1-hESC	CL:000003429
ENCSR490SQH	ENCODE	H7-hESC	CL:000003429
C0066P12	Blueprint	CD8-positive, alpha-beta T cell	CL:00006255
C005PS12	Blueprint	CD14-positive, CD16-negative classical monocyte	CL:00020571427
S00DFM11	Blueprint	Acute Lymphocytic Leukemia	CL:00020921467
S00HSH11	Blueprint	macrophage - T=6days LPS	CL:0000235209
S00JRB11	Blueprint	macrophage - T=6days LPS	CL:0000235209
S00BYT11	Blueprint	macrophage - T=6days LPS	CL:0000235209
S00CS011	Blueprint	macrophage - T=6days LPS	CL:0000235209
C006NSB1	Blueprint	CD34-negative, CD41-positive, CD42-positive megakaryocyte cell	CL:00020051368
S004BT	Blueprint	CD34-negative, CD41-positive, CD42-positive megakaryocyte cell	CL:00020051368
S008H111	Blueprint	CD4-positive, alpha-beta T cell	CL:0000624532
S002R512	Blueprint	erythroblast	CL:0000765677
S002S312	Blueprint	erythroblast	CL:0000765677
S001S714	Blueprint	Macrophage	CL:0000235209
S001MJ12	Blueprint	Inflammatory macrophage	CL:0000863793
S0022I14	Blueprint	Inflammatory macrophage	CL:0000863793
S00HRJ11	Blueprint	macrophage - T=6days untreated	CL:0000235209
S00BXV11	Blueprint	macrophage - T=6days untreated	CL:0000235209
S00CR211	Blueprint	macrophage - T=6days untreated	CL:0000235209
S00JQD11	Blueprint	macrophage - T=6days untreated	CL:0000235209
S013M311	Blueprint	Acute Myeloid Leukemia	CL:0000766678
S00D0F11	Blueprint	Acute Myeloid Leukemia	CL:0000766678
S00D6311	Blueprint	Acute Myeloid Leukemia	CL:0000766678
S005EJ11	Blueprint	Acute Myeloid Leukemia	CL:0000766678
S013QW11	Blueprint	Acute Myeloid Leukemia	CL:0000766678
S005FH11	Blueprint	Acute Myeloid Leukemia	CL:0000766678
S00XXH11	Blueprint	Acute Myeloid Leukemia	CL:0000766678
S00CXR11	Blueprint	Acute Myeloid Leukemia	CL:0000766678
S013N111	Blueprint	Acute Myeloid Leukemia	CL:0000766678
S00CYP11	Blueprint	Acute Myeloid Leukemia	CL:0000766678
S00D5511	Blueprint	Acute Myeloid Leukemia	CL:0000766678
S00D3911	Blueprint	Acute Myeloid Leukemia	CL:0000766678
S00XUN11	Blueprint	Acute Myeloid Leukemia	CL:0000766678
S00XYF11	Blueprint	Acute Myeloid Leukemia	CL:0000766678
S013PY11	Blueprint	Acute Myeloid Leukemia	CL:0000766678
S00Y1311	Blueprint	Acute Myeloid Leukemia	CL:0000766678
S00XWJ11	Blueprint	Acute Myeloid Leukemia	CL:0000766678
S013RU11	Blueprint	Acute Myeloid Leukemia	CL:0000766678
S00D1D11	Blueprint	Acute Myeloid Leukemia	CL:0000766678
S00Y0511	Blueprint	Acute Myeloid Leukemia	CL:0000766678
S00D4711	Blueprint	Acute Myeloid Leukemia	CL:0000766678
S00XVL11	Blueprint	Acute Myeloid Leukemia	CL:0000766678
S013SS11	Blueprint	Acute Myeloid Leukemia	CL:0000766678
S00Y6U11	Blueprint	Acute Myeloid Leukemia	CL:0000766678
S00CWT11	Blueprint	Acute Myeloid Leukemia	CL:0000766678
S00Y4Y11	Blueprint	Acute Myeloid Leukemia	CL:0000766678
S0022I12	Blueprint	Macrophage	CL:0000235209

C005VG11	Blueprint	Macrophage	CL:0000235209
S00B0N11	Blueprint	Chronic Lymphocytic Leukemia	CL:00025431948
S00B2J11	Blueprint	Chronic Lymphocytic Leukemia	CL:00025431948
S00C0J11	Blueprint	macrophage - T=6days B-glucan	CL:0000235209
S00HTF11	Blueprint	macrophage - T=6days B-glucan	CL:0000235209
S00JS911	Blueprint	macrophage - T=6days B-glucan	CL:0000235209
S00CTZ11	Blueprint	macrophage - T=6days B-glucan	CL:0000235209
C0010KB1	Blueprint	CD14-positive, CD16-negative classical monocyte	CL:00010541126
C001UYB4	Blueprint	CD14-positive, CD16-negative classical monocyte	CL:00010541126
C0011IB1	Blueprint	CD14-positive, CD16-negative classical monocyte	CL:00010541126
ENCSR637GBV	Roadmap	Skin fibroblast	CL:00026202024
ENCSR655XQF	Roadmap	Skin fibroblast	CL:00026202024
ENCSR022MON	Roadmap	Skin fibroblast	CL:00026202024
ENCSR982VYI	Roadmap	Skin fibroblast	CL:00026202024
ENCSR361DRG	Roadmap	Fibroblast of skin of abdomen	CL:20000131190
ENCSR681ALA	Roadmap	Fibroblast of skin of abdomen	CL:20000131190
ENCSR762CJN	Roadmap	Trophoblast cell	CL:0000351287
ENCSR406YML	Roadmap	Muscle of arm	CL:0000188171
ENCSR364IBB	Roadmap	Muscle of arm	CL:00001881
ENCSR317LMH	Roadmap	Muscle of arm	CL:00001881
ENCSR620ZNQ	Roadmap	Muscle of arm	CL:00001881
ENCSR305NXN	Roadmap	Muscle of arm	CL:00001881
ENCSR677MYO	Roadmap	Muscle of arm	CL:00001881
ENCSR990LHE	Roadmap	Muscle of arm	CL:00001881
ENCSR922VBO	Roadmap	Stomach	CL:1000313832
ENCSR721HDG	Roadmap	Stomach	CL:1000313832
ENCSR702IGQ	Roadmap	Stomach	CL:1000313832
ENCSR549DVY	Roadmap	Stomach	CL:1000313832
ENCSR783BUO	Roadmap	Stomach	CL:1000313832
ENCSR951NPS	Roadmap	Stomach	CL:1000313832
ENCSR123ZCX	Roadmap	Stomach	CL:1000313832
ENCSR774SEX	Roadmap	Stomach	CL:1000313832
ENCSR729ZII	Roadmap	Muscle of back	CL:0000188171
ENCSR806ESH	Roadmap	Muscle of back	CL:0000188171
ENCSR995ORR	Roadmap	Muscle of back	CL:0000188171
ENCSR891JVD	Roadmap	Muscle of back	CL:0000188171
ENCSR652AWW	Roadmap	Muscle of back	CL:0000188171
ENCSR027EJD	Roadmap	Muscle of back	CL:0000188171
ENCSR576UKA	Roadmap	Muscle of back	CL:0000188171
ENCSR094RGI	Roadmap	Muscle of back	CL:0000188171
ENCSR239BBI	Roadmap	Muscle of back	CL:0000188171
ENCSR522XTV	Roadmap	Muscle of back	CL:0000188171
ENCSR719HRO	Roadmap	Small intestine	CL:10015981554
ENCSR621FYE	Roadmap	Small intestine	CL:10015981554
ENCSR150JIX	Roadmap	Small intestine	CL:10015981554
ENCSR446RKD	Roadmap	Small intestine	CL:10015981554
ENCSR523EDD	Roadmap	Small intestine	CL:10015981554
ENCSR096USV	Roadmap	Muscle of leg	CL:0000188171
ENCSR860DST	Roadmap	Muscle of leg	CL:0000188171

ENCSR144UVO	Roadmap	Muscle of leg	CL:0000188171
ENCSR545WAC	Roadmap	Muscle of leg	CL:0000188171
ENCSR174ESD	Roadmap	Muscle of leg	CL:0000188171
ENCSR086DZF	Roadmap	Muscle of leg	CL:0000188171
ENCSR561WEX	Roadmap	Muscle of leg	CL:0000188171
ENCSR447UE	Roadmap	Muscle of leg	CL:0000188171
ENCSR286KWP	Roadmap	Large intestine	CL:1000320881
ENCSR859KGW	Roadmap	Large intestine	CL:1000320881
ENCSR777ONH	Roadmap	Large intestine	CL:1000320881
ENCSR930URM	Roadmap	Large intestine	CL:1000320881
ENCSR857VKL	Roadmap	Large intestine	CL:1000320881
ENCSR363BVC	Roadmap	Large intestine	CL:1000320881
ENCSR861SOG	Roadmap	Left lung	CL:00020621433
ENCSR733MWN	Roadmap	Left lung	CL:00020621433
ENCSR592EZK	Roadmap	Left lung	CL:00020621433
ENCSR499NEL	Roadmap	Left lung	CL:00020621433
ENCSR222IGR	Roadmap	Left lung	CL:00020621433
ENCSR572FXC	Roadmap	Left lung	CL:00020621433
ENCSR907KDH	Roadmap	Kidney	CL:10004971165
ENCSR212AMA	Roadmap	Kidney	CL:10004971165
ENCSR896QPD	Roadmap	Kidney	CL:10004971165
ENCSR495UXA	Roadmap	Kidney	CL:10004971165
ENCSR554KBK	Roadmap	Right lung	CL:00020621433
ENCSR074APH	Roadmap	Right lung	CL:00020621433
ENCSR560MDQ	Roadmap	Right lung	CL:00020621433
ENCSR176WMG	Roadmap	Right lung	CL:00020621433
ENCSR044JAQ	Roadmap	Right lung	CL:00020621433
ENCSR367QHR	Roadmap	Thymus	CL:00022931702
ENCSR158XIJ	Roadmap	Thymus	CL:00022931702
ENCSR069CMT	Roadmap	Thymus	CL:00022931702
ENCSR175CNQ	Roadmap	Thymus	CL:00022931702
ENCSR047LIJ	Roadmap	Heart	CL:00024941900
ENCSR863BUL	Roadmap	Heart	CL:00024941900
ENCSR328PVI	Roadmap	Renal cortex interstitium	CL:10005961200
ENCSR899SWV	Roadmap	Renal cortex interstitium	CL:10005961200
ENCSR436ZKE	Roadmap	Renal cortex interstitium	CL:10005961200
ENCSR335GET	Roadmap	Adrenal gland	CL:10016011602
ENCSR120NEA	Roadmap	Adrenal gland	CL:10016011602
ENCSR688YOZ	Roadmap	Adrenal gland	CL:10016011602
ENCSR7400OPV	Roadmap	Adrenal gland	CL:10016011602
ENCSR424TSZ	Roadmap	Renal Pelvis	CL:10004971165
ENCSR204XBB	Roadmap	Renal Pelvis	CL:10004971165
ENCSR929KRW	Roadmap	Renal Pelvis	CL:10004971165
ENCSR702IMR	Roadmap	Left Kidney	CL:10004971165
ENCSR015EMF	Roadmap	Left renal cortex interstitium	CL:10005961200
ENCSR125NGM	Roadmap	Left renal cortex interstitium	CL:10005961200
ENCSR759WPF	Roadmap	Left renal cortex interstitium	CL:10005961200
ENCSR413LXW	Roadmap	Left renal cortex interstitium	CL:10005961200
ENCSR029FTY	Roadmap	Left renal pelvis	CL:10004971165
ENCSR321ROU	Roadmap	Left renal pelvis	CL:10004971165
ENCSR410DUZ	Roadmap	Left renal pelvis	CL:10004971165
ENCSR160UAZ	Roadmap	Left renal pelvis	CL:10004971165

ENCSR552YAE	Roadmap	Right renal pelvis	CL:10004971165
ENCSR352GCS	Roadmap	Right renal pelvis	CL:10004971165
ENCSR543TQW	Roadmap	Right renal pelvis	CL:10004971165
ENCSR928CEQ	Roadmap	Right renal pelvis	CL:10004971165
ENCSR899NLW	Roadmap	Spinal cord	CL:00050002081
ENCSR333FZW	Roadmap	Spinal cord	CL:00050002081
ENCSR822AOE	Roadmap	Right renal cortex interstitium	CL:10005961200
ENCSR884EVS	Roadmap	Right renal cortex interstitium	CL:10005961200
ENCSR400DJE	Roadmap	Right renal cortex interstitium	CL:10005961200
ENCSR265NZF	Roadmap	Spleen	CL:000265120
ENCSR817TLH	Roadmap	Psoas muscle	CL:0000188171
ENCSR531RKI	Roadmap	Muscle of trunk	CL:0000188171
ENCSR727VTD	Roadmap	Ovary	CL:00020941469
ENCSR725TPW	Roadmap	Ovary	CL:00020941469
ENCSR629VMZ	Roadmap	Pancreas	CL:10015991552
ENCSR571BML	Roadmap	Pancreas	CL:10015991552
ENCSR755LFM	Roadmap	Testis	CL:00022381649
ENCSR711NGL	Roadmap	Forelimb muscle	CL:0000188171
ENCSR516VDS	Roadmap	Hindlimb muscle	CL:0000188171
ENCSR911GQI	Roadmap	H1-hESC	CL:000003429
ENCSR844HLP	Roadmap	H1-hESC	CL:000003429

Supplementary Table 1: Sample IDs (DEEP and Blueprint)/ Experiment IDs(ENCODE, Roadmap) are listed with the assigned tissue, the related consortium, and mapped Cell Ontology Terms. Note that Roadmap samples originating from the different biosamples are considered as biological replicates. The biosample IDs can be accessed via the Experiment IDs provided here.

Consortia	Counts
DEEP	12
ENCODE	36
Blueprint	56
Roadmap	112

Supplementary Table 2: Sample Counts per Consortium for IHEC data

Tissue	Consortia	Counts	Cell Ontology Term
Thyroid	TCGA	59	CL:0000452
Thyroid	GTEx	355	CL:0000452
Liver	TCGA	50	CL:0000182
Liver	GTEx	176	CL:0000182
Kidney	TCGA	72	CL:1000497
Kidney	GTEx	36	CL:1000497
Colon	TCGA	41	CL:1001588
Colon	GTEx	376	CL:1001588
Prostate	TCGA	52	CL:0002231
Prostate	GTEx	119	CL:0002231

Supplementary Table 3: Sample Counts per Tissue, Consortium and Cell Ontology Terms for GTex and TCGA data

2. Results

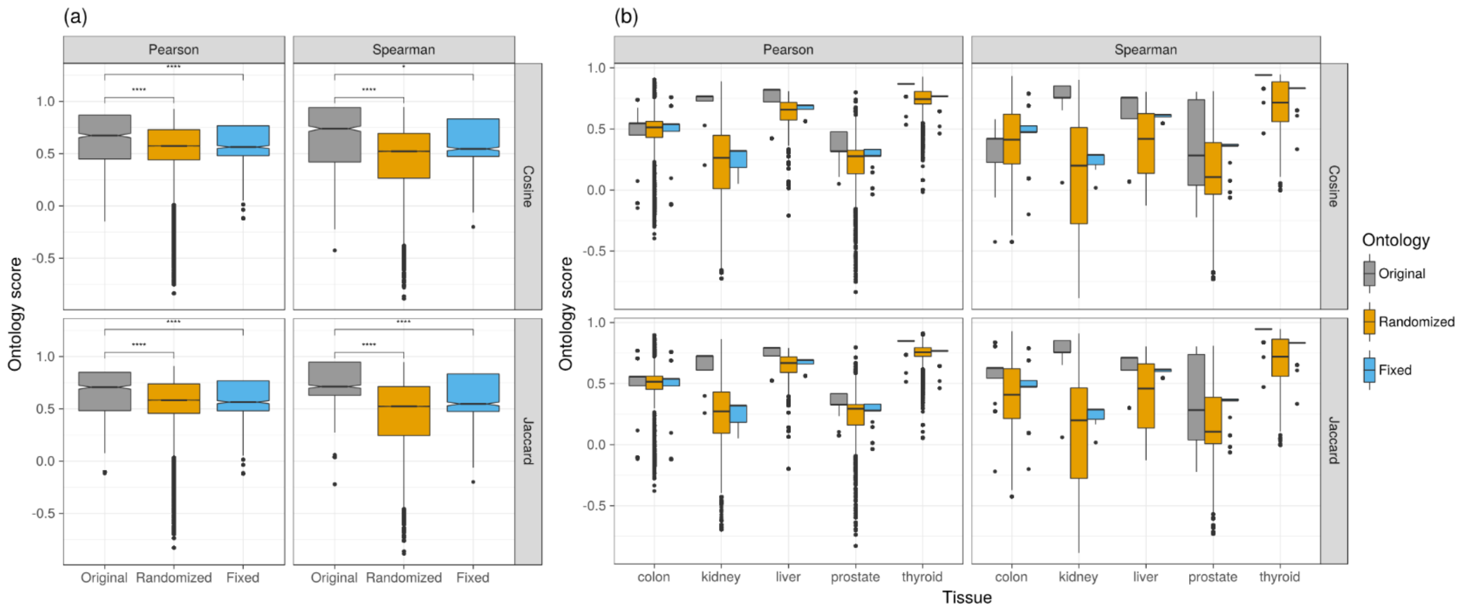

Supplementary Figure 1: a) Shows the ontology score computed using the original ontology mapping, a fixed ontology assignment (1.0 for samples from the same tissue, 0.25 otherwise), and randomly sampled ontologies. For all score variations, the difference between the original ontology score and the alternative versions is significant (Wilcoxon Man-Whitney Test) **b)** Shows the same data as a) but split into individual tissues.

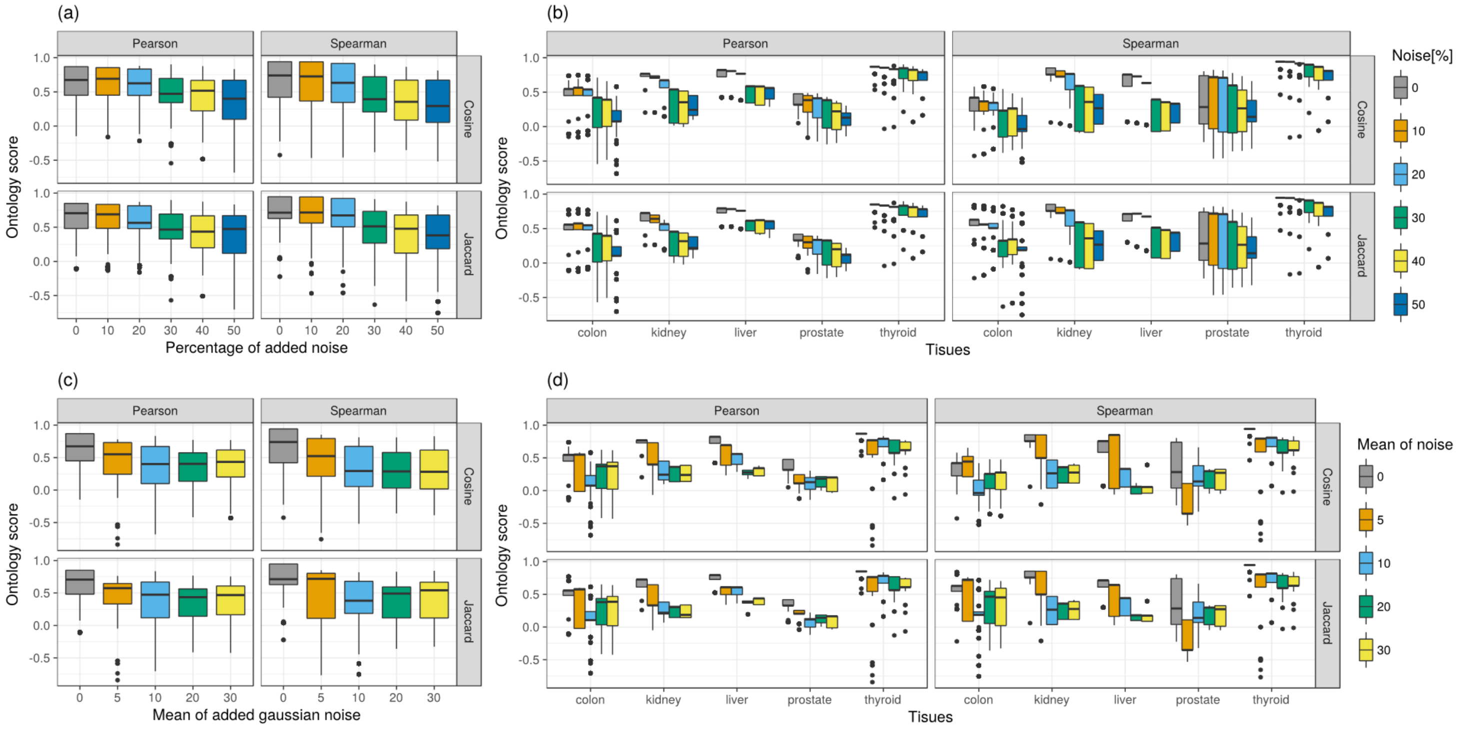

Supplementary Figure 2: a) Ontology score depending on the percentage of samples with added Gaussian noise. One can see that the score is declining for all four score variants. **b)** Shows the same as a) but for individual tissues. . **c)** Ontology score depending on the mean of added Gaussian noise. Except for the score combination Spearman and Jaccard, one can see that the score drops with increasing mean and stays roughly constant after the mean is larger than ten. **d)** Shows the same as c) but zoomed on the tissue level.

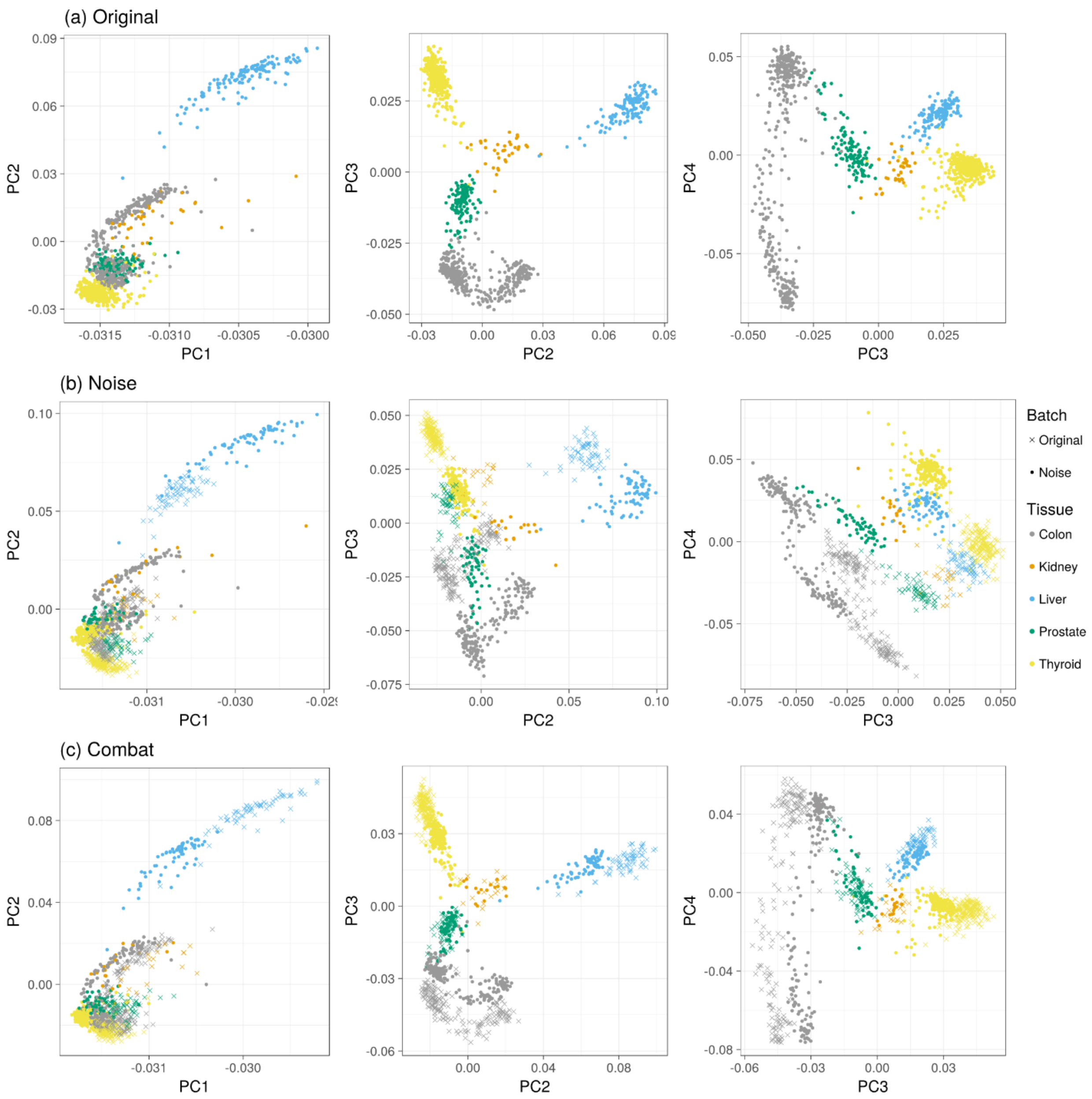

Supplementary Figure 3: This Figure shows the results of a PCA analysis (PC1-PC4) for three different scenarios: **a)** Original GTEx data before noise addition. **b)** Data after adding Gaussian noise with mean 10 and standard deviation 1 on 50% of samples per tissue **c)** Data after correcting using Combat. One can see that the shift introduced in b is removed through combat for PC2, PC3 and PC4. However, it is still visible in the liver samples for PC1.

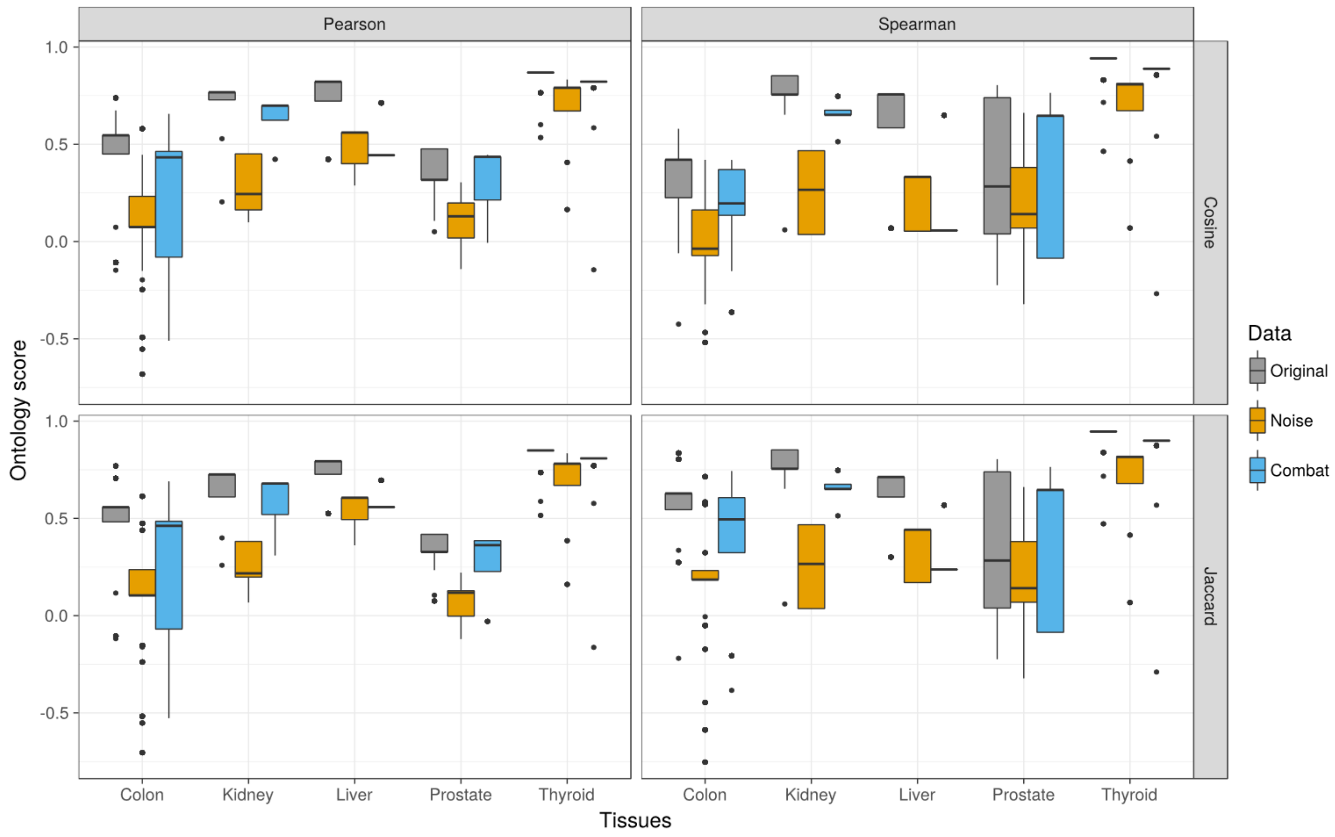

Supplementary Figure 4: Ontology score for individual GTEx tissues before noise addition (Original), after Noise addition (Orange) and after noise removal with Combat (blue). One can see that only for liver samples, Combat is not able to restore the ontology score. For the remaining tissues, combat is able to adequately remove the artificial noise.

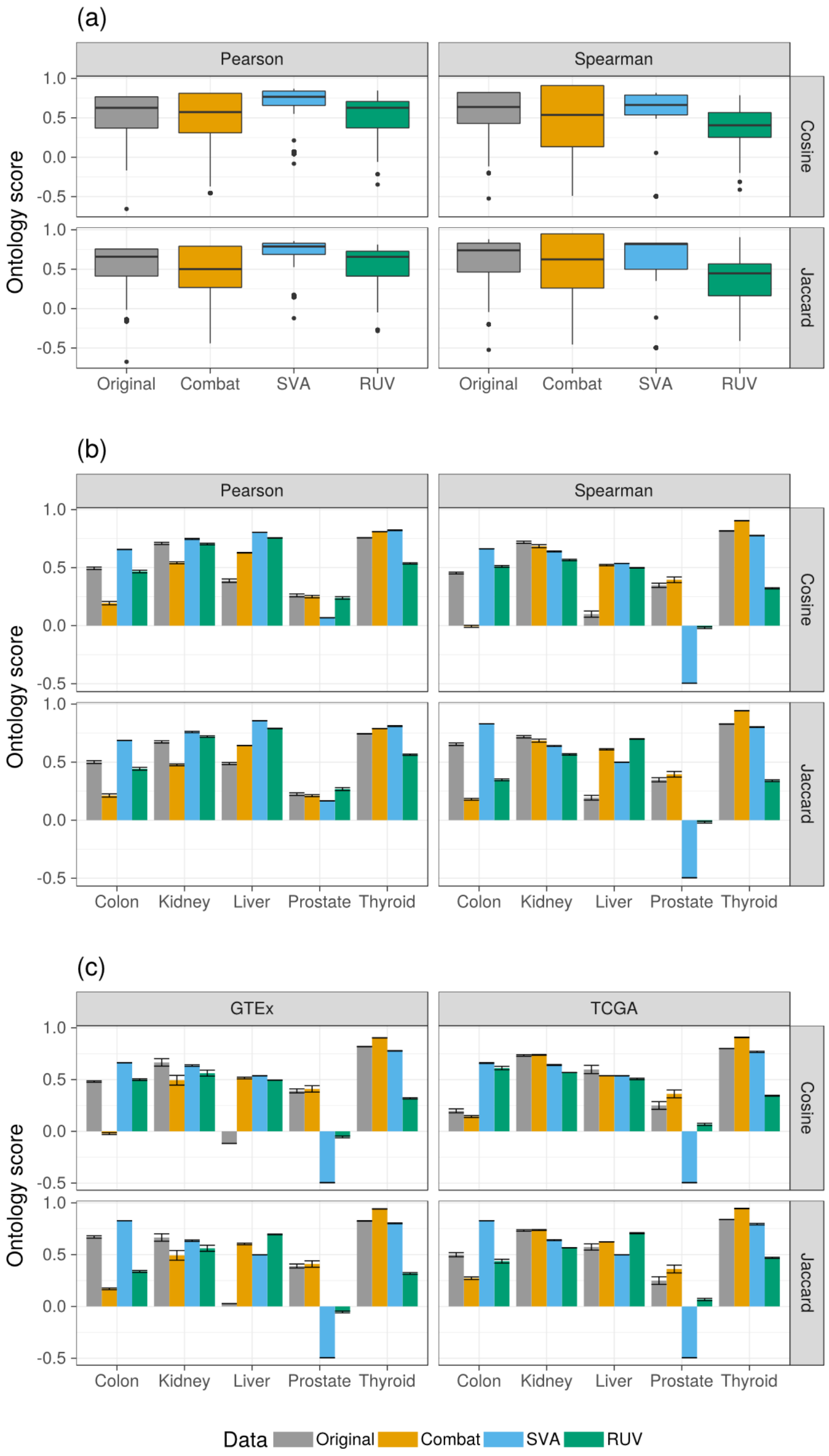

Supplementary Figure 5: a) Ontology score for the TCGA/GTEx scenario in all score variants. According to Pearson correlation using SVA improves the score slightly, while this cannot be seen in Spearman correlation. To understand this difference, the tissue specific plots in **b)** can be used. As one can see, the score for Prostate is largely negative using Spearman correlation, which affects the median strongly, those no beneficial effect can be seen on average. **c)** Here, the scores are depicted depending on tissue and consortia of origin. One can see that prostate samples cannot be corrected well in both consortia, whereas liver data is significantly boosted in GTEx but not in TCGA data. This explains why SVA performs better for GTEX data on average than for TCGA data (Fig. 5a).

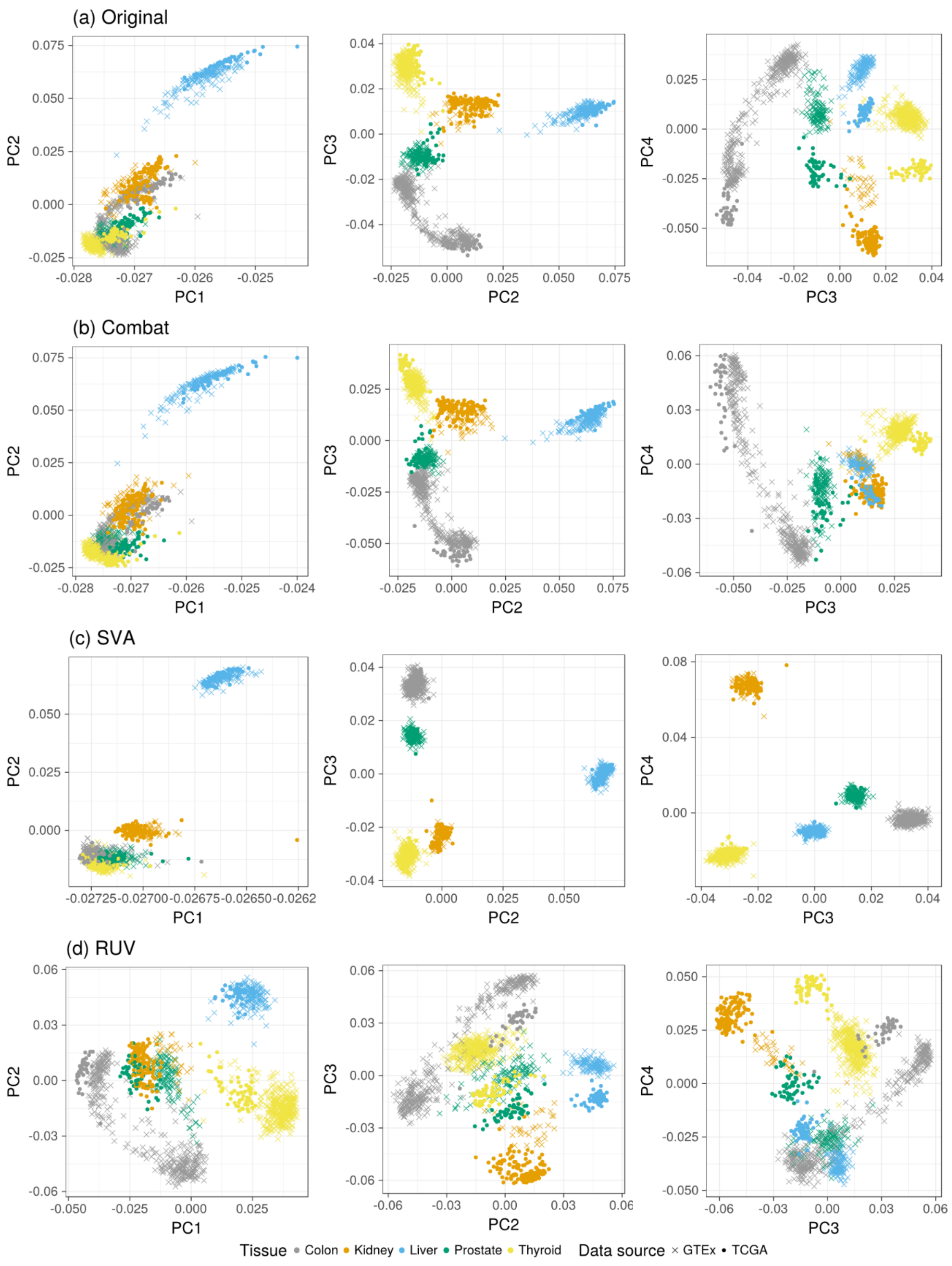

Supplementary Figure 6: PCA analysis showing PC1-PC4 for the TCGA/GTEX experiment **a)** Original TCGA and GTEx data. **b)** Data after Combat normalization. **c)** SVA normalization **d**) RUV normalization. Although SVA seems to perform very well in PC2, PC3, and PC4, it seems that the cluster generated in PC1 is not meaningful and affects the ontology score, as shown in Sup. Fig 5, in a negative way for the liver samples.

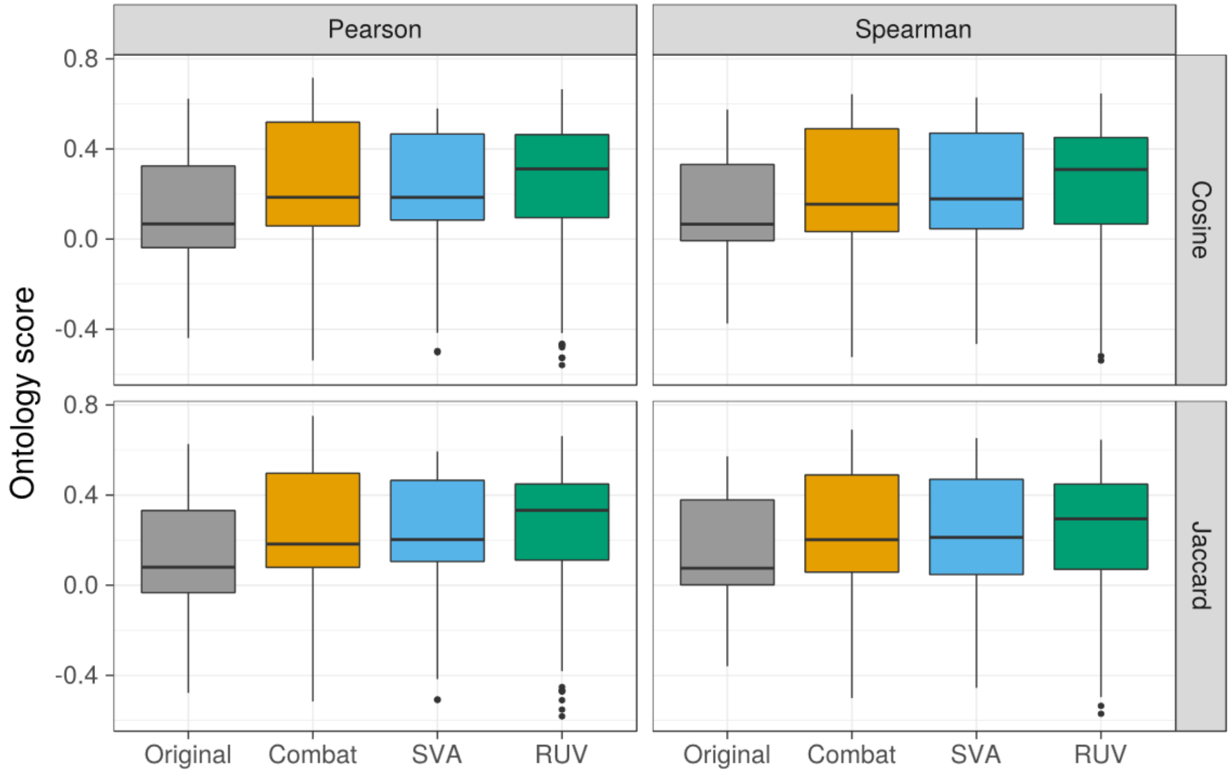

Supplementary Figure 7: General performance of Combat, SVA, and RUV on the IHEC data set. On average, the RUV method obtains the highest ontology scores across all four score combinations.

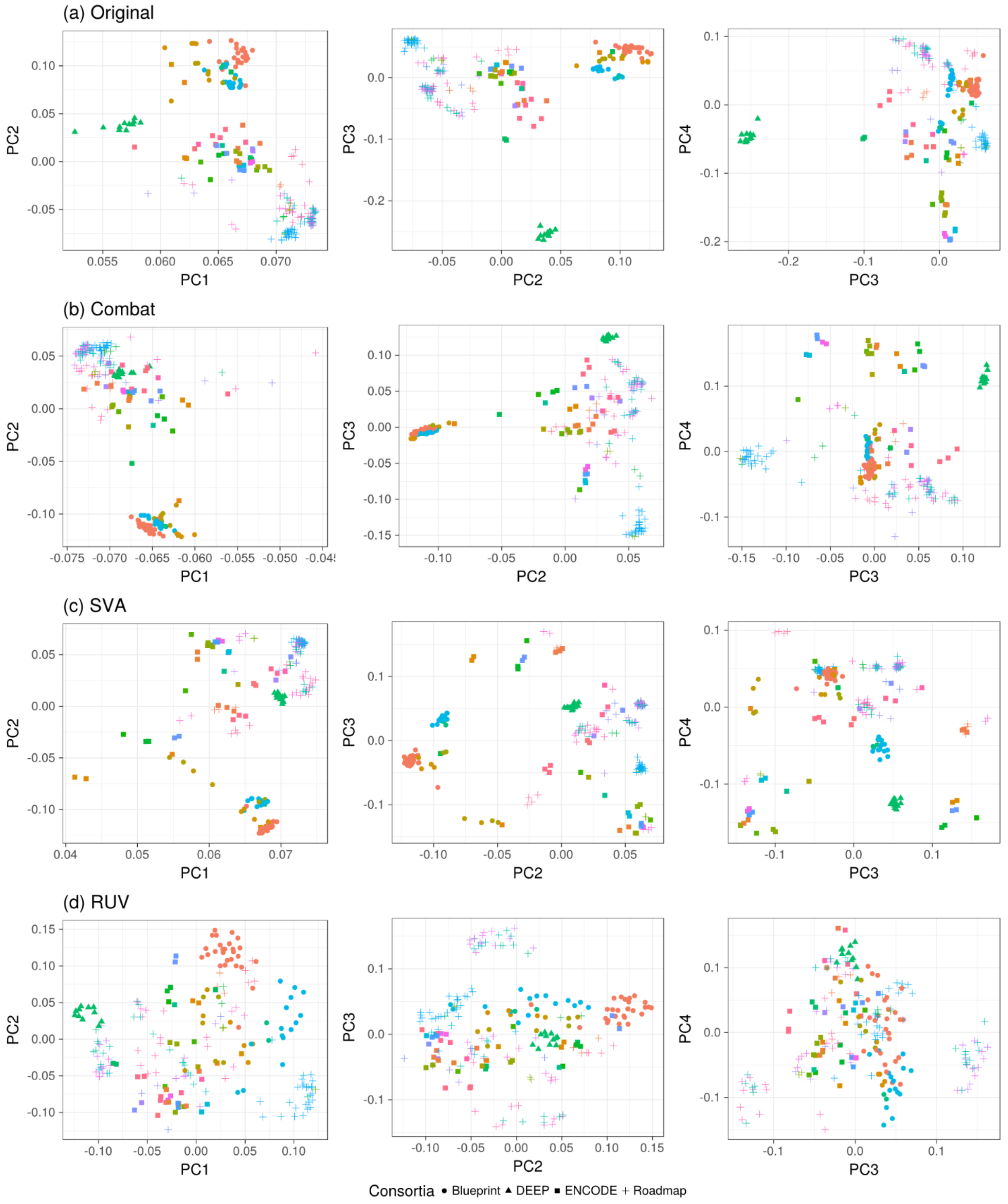

Supplementary Figure 8: PCA analysis showing PC1-PC4 for the IHEC experiment **a)** Original TCGA and GTEx data. **b)** Data after Combat normalization. **c)** SVA normalization **d**) RUV normalization. Here, the RUV normalization generates a clustering that tends to represent rather tissue similarities than consortia effects.

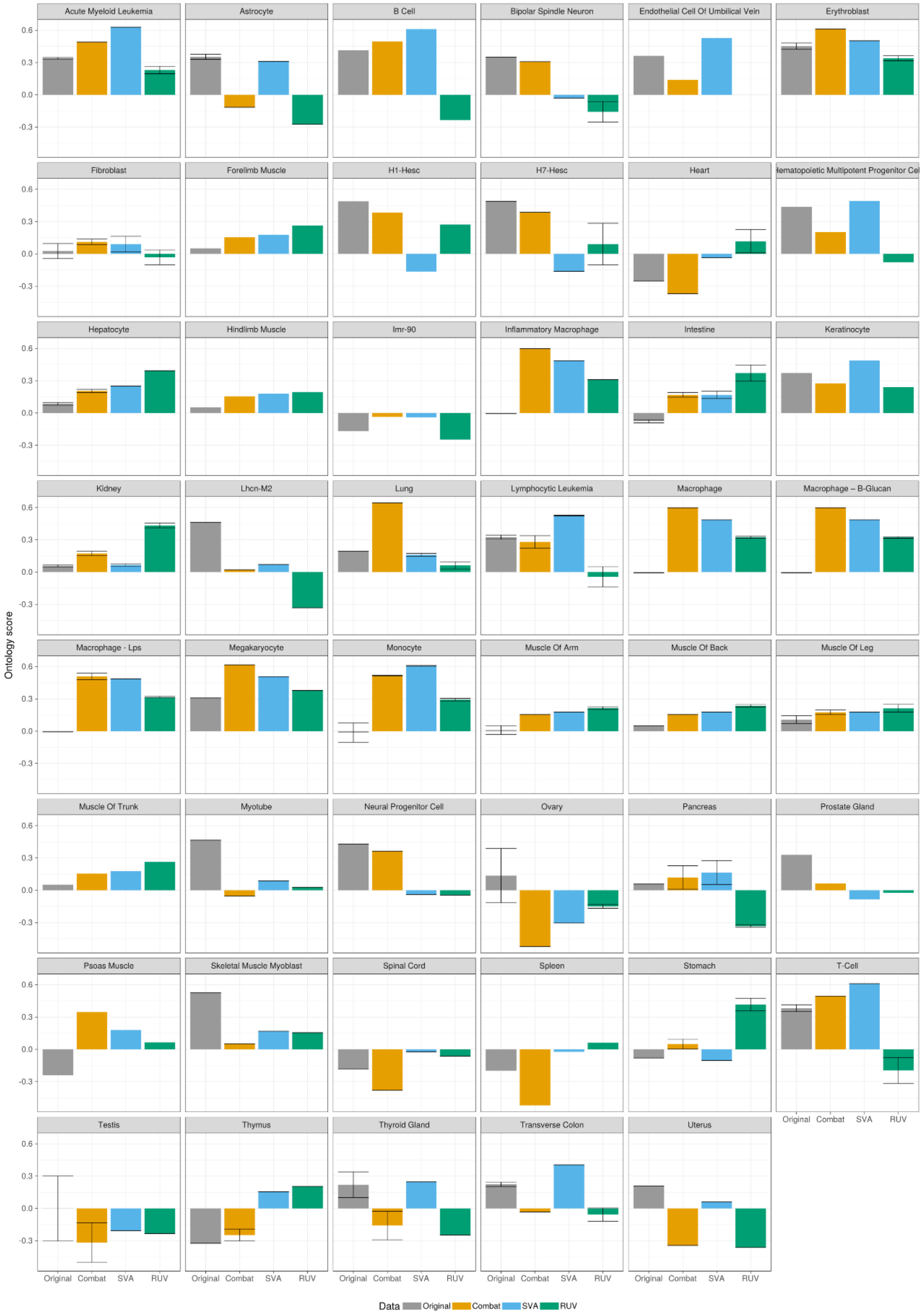

Supplementary Figure 9: Ontology score shown for all tissues of the IHEC data set and depending on the used data normalization method. One can see that no method is generally applicable across all tissues. Although the RUV method performs favorably in most cases. Here, we show Spearman correlation with Cosine similarity. The same observations can be made using the remaining score variants.

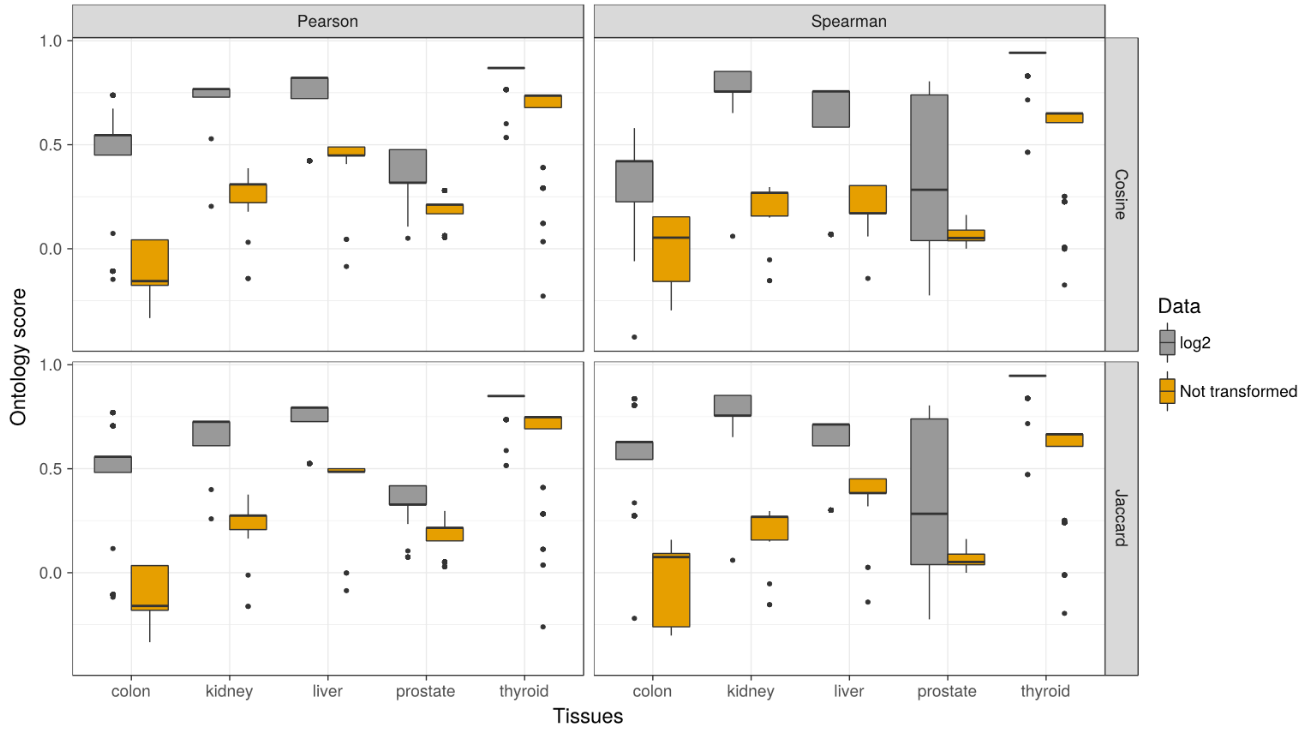

Supplementary Figure 10: Ontology score for GTEx data illustrating the effect of a log2 transformation of the read counts after applying Deseq2 normalization.

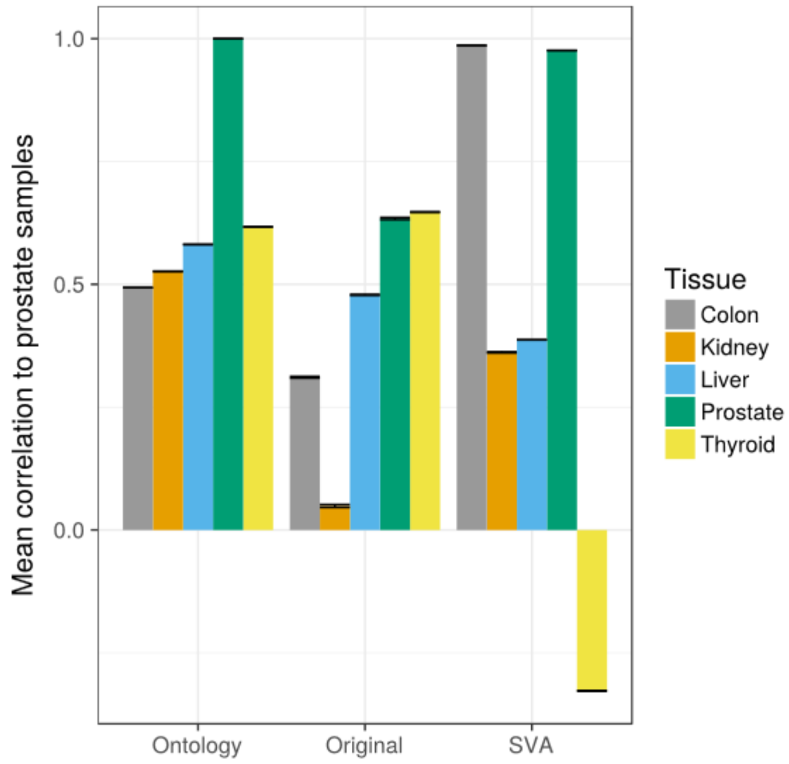

Supplementary Figure 11: Spearman correlation of prostate samples among each other computed for the original and SVA corrected data as well as for the Cell Ontology. In the SVA corrected data, the prostate samples are negatively correlated to thyroid and obtain a high correlation to the colon samples.

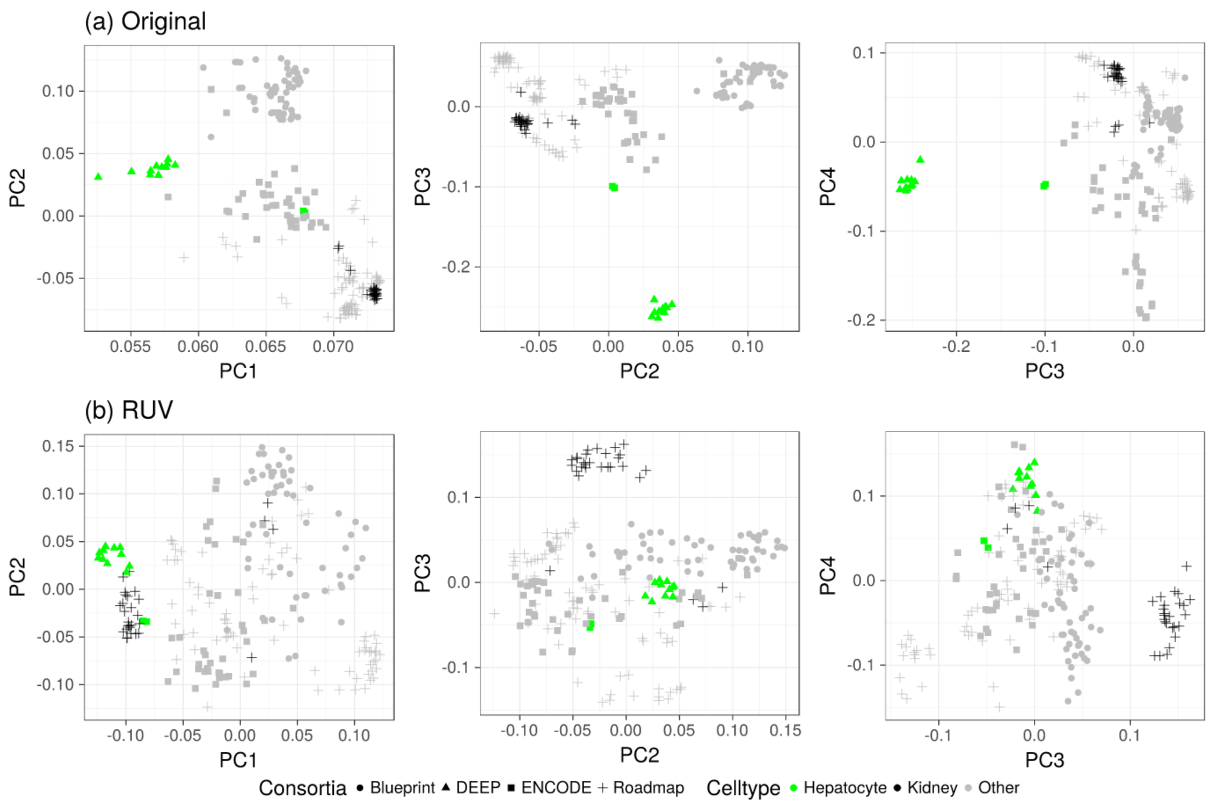

Supplementary Figure 12: PCA analysis comparing original (a) gene expression data against RUV normalized data (b). Highlighted are hepatocyte and kidney samples. One can see (1) that DEEP hepatocyte samples cluster closer to ENCODE hepatocytes and (2) kidney samples now form a separate cluster, detached from the remaining Roadmap samples.

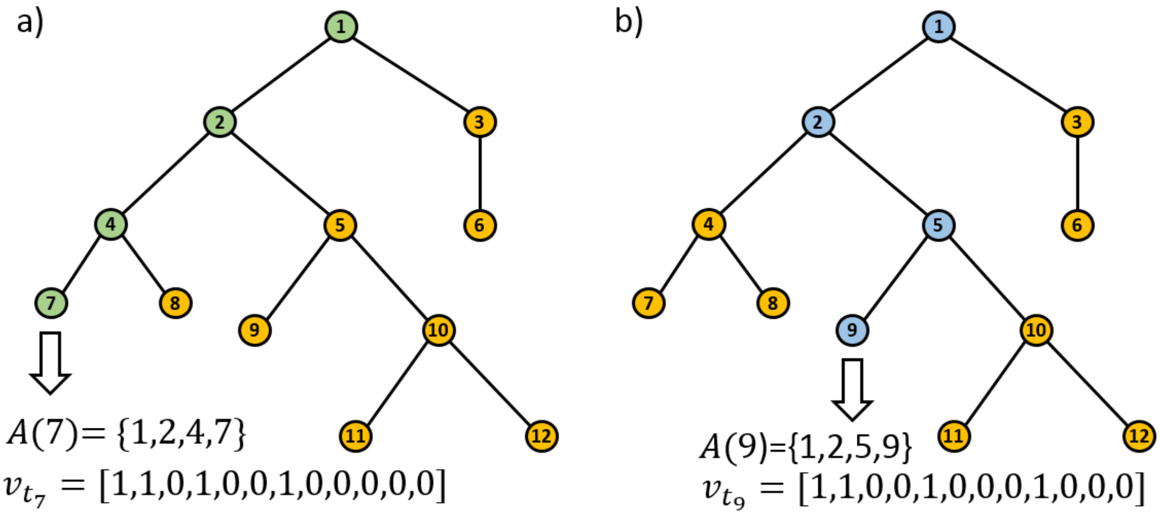

c)

$$sim_{jac}(t_7, t_9) = \frac{|A(t_7) \cap A(t_9)|}{|A(t_7) \cup A(t_9)|} = \frac{|\{1,2,4,7\} \cap \{1,2,5,9\}|}{|\{1,2,4,7\} \cup \{1,2,5,9\}|} = \frac{|\{1,2\}|}{|\{1,2,4,5,7,9\}|} = \frac{1}{3}$$

d)

$$sim_{cos}(t_7, t_9) = \frac{v_{t_7} \cdot v_{t_9}}{\sqrt{v_{t_7}^2} \cdot \sqrt{v_{t_9}^2}} = \frac{\sum_{i=1}(v_{t_{7}{}_i} \cdot v_{t_{9}{}_i})}{\sqrt{\sum_{i=1}(v_{t_{7}{}_i})^2} \cdot \sqrt{\sum_{i=1}(v_{t_{9}{}_i})^2}} = \frac{2}{2 * 2} = \frac{1}{2}$$

Supplementary Figure 13: Example for the computation of the expected similarity. We show the successor for term 7 and term 9, as well as the vector representations for term 7 and 9 in (a) and (b) respectively. An example for the computation of the Jaccard similarity is shown in (c), and an example for the cosine similarity is shown in (d).
